# Supplementary material for: Opportunities and challenges of a novel cardiac output response to stress (CORS) test to enhance diagnosis of heart failure in primary care: qualitative study
Source: BMJ Open. 2019 Apr 14;9(4):e028122. doi: 10.1136/bmjopen-2018-028122 (PMC6500186; doi:10.1136/bmjopen-2018-028122)
Supplement: Supplementary appendix [file bmjopen-2018-028122supp001.pdf]

Interview Schedule v1: Consultant Cardiologists
Project Title: Confirmation of Acceptability of a Novel Cardiac Output Response to Stress (CORS) Test to Improve Diagnosis of Heart Failure in Primary Care

PART 1

CLINICAL CARE PATHWAY

What is the clinical care pathway for a patient with a suspected heart failure diagnosis in primary care?

When is a patient referred to secondary care?

Do the referral times from suspected heart failure diagnosis in primary care to secondary care need to be improved?

Prompt: if so, what needs to be improved? Strategy for improvement

What is your role in the clinical care pathway for a patient with a suspected heart failure diagnosis?

During a heart failure diagnostic clinic, how many suspected cases of heart failure do you typically see?

How many of these cases lead to diagnosis of heart failure?

Does the current clinical care pathway lead to you seeing many patients who are unlikely to receive a heart failure diagnosis in secondary care?

Prompt: If so how can this be issue be addressed?

How long does it take to determine a suspected heart failure case in secondary care?

Do the referral times from suspected heart failure diagnosis in primary care to confirmed diagnosis need to be improved?

Prompt: if so what needs to change?

VIDEO OF CARDIAC OUTPUT RESPONSE TO STRESS (CORS) TEST

We are proposing a novel test which has been demonstrated to improve suspected heart failure diagnosis. This should therefore improve the number of patient referrals to secondary care.

PART 2

NEW CORS TEST

This novel test has been demonstrated to improve suspected heart failure diagnosis. This should therefore improve the number of patient referrals to secondary care.

What are your thoughts about this potential improvement in diagnosis being implemented in the clinical care pathway for heart failure?

Prompt: design, suitability and access to patients

What is your understanding of the new test?

Was there any information in the demonstration video that was difficult to understand?

Would a patient with a suspected heart failure diagnosis be able to complete this test?

Prompt: possible challenges to patients, Physical ability, other health problems

Where in the clinical care pathway for suspected heart failure diagnosis would you expect this test to be performed?

Would you expect this test to be performed in primary or secondary care?

Prompt: advantages/disadvantages; access to test, costs, practical implications

If this test was to become a routine part of clinical care for a patient with a suspected heart failure diagnosis, who would you expect to deliver it in primary care?

Prompt: rational for opinion

If this test was to become a routine part of clinical care for a patient with a suspected heart failure diagnosis, who would you expect to deliver it in secondary care?

Prompt: rational for opinion

Who has the greater capacity to run this test as a routine part of clinical care for a patient with a suspected heart failure diagnosis?

What problems or difficulties do you think a healthcare professional (e.g. practice nurse) running this test may encounter with a patient performing this test?

What else might be needed to deliver this test in either primary or secondary care?

What training would be needed to be able to run this test efficiently?

Do you have any further comments about running this test as part of your routine clinical care for a patient with a suspected heart failure diagnosis?

Interview Schedule v1: General Practitioners (Primary Care)

Project Title: Confirmation of Acceptability of a Novel Cardiac Output Response to Stress (CORS) Test to Improve Diagnosis of Heart Failure in Primary Care

PART 1

CLINICAL CARE PATHWAY

What is the clinical care pathway for a patient with a suspected heart failure diagnosis in primary care?

What is your role in the clinical care pathway for a patient with a suspected heart failure diagnosis?

What tests are performed to determine risk of heart failure?

Who would perform these tests in your practice?

How many times is a patient seen before they are referred to secondary care with suspected heart failure diagnosis?

How long is a typical consultation with a patient with a suspected heart failure diagnosis?

Who would inform a patient who was on the clinical care pathway for suspected heart failure diagnosis?

REFERRAL

When is a patient referred to secondary care?

What is your role in the referral process?

What are your thoughts on the referral time to secondary care?

When do you expect to receive the outcome from the referral?

What are the next steps after a patient has been diagnosed with heart failure?

What is your role in the continuing care of a patient diagnosed with heart failure?

Do the referral times from suspected heart failure diagnosis in primary care to confirmed diagnosis need to be improved?

Prompt: what needs improving? Strategy for improvement?

VIDEO OF CARDIAC OUTPUT RESPONSE TO STRESS (CORS) TEST

We are proposing a novel test which has been demonstrated to improve suspected heart failure diagnosis. This should therefore improve the number of patient referrals to secondary care

PART 2

NEW CORS TEST

This novel test has been demonstrated to improve suspected heart failure diagnosis. This should therefore improve the number of patient referrals to secondary care.

What are your thoughts about this potential improvement in diagnosis being implemented in the clinical care pathway for heart failure?

What is your understanding of the new test?

Was there any information in the demonstration video that was difficult to understand?

Would a patient with a suspected heart failure diagnosis be able to complete this test?

Could this test be added into a routine consultation for suspected heart failure diagnosis? Or would you expect the patient need to attend a separate appointment to complete the test?

Do you anticipate any immediate barriers to implementing this test as part of your routine clinical care for a patient with a suspected heart failure diagnosis in your practice?

Prompt: How easy would it be to implement this test in your practice? Are there challenges?

If this test was to become a routine part of clinical care for a patient with a suspected heart failure diagnosis, who would you expect to deliver it?

Prompt: rational for opinion

What problems or difficulties do you think you (or the nominated colleague, i.e. practice nurse) may encounter when using this test?

What else might you need to deliver this test in practice?

Prompt: Training needs

How likely is it that this test will become part of routine clinical care for a patient with a suspected heart failure diagnosis?

Prompt: Diagnostic urgency, suitability for patients, convenience

Where in the clinical care pathway for suspected heart failure diagnosis would you anticipate this test being implemented?

Prompt: Rational for opinion

Where do you think this test would be delivered in your practice?

Prompt: rational for opinion

Do you have the capacity to run this test as a routine part of clinical care for suspected heart failure diagnosis?

Do you have any further comments about running this test as part of your routine clinical care for a patient with a suspected heart failure diagnosis?

Interview Schedule v1: Practice Nurse (Primary Care)

Project Title: Confirmation of Acceptability of a Novel Cardiac Output Response to Stress (CORS) Test to Improve Diagnosis of Heart Failure in Primary Care

PART 1

CLINICAL CARE PATHWAY

What is the clinical care pathway for a patient with a suspected heart failure diagnosis in primary care?

What is your role in the clinical care pathway for a patient with a suspected heart failure diagnosis?

What tests are performed to determine risk of heart failure?

Who would perform these tests in your practice?

How long do the tests typically take?

How many times is a patient seen before they are referred to secondary care with a suspected heart failure diagnosis?

How long is a typical suspected heart failure diagnosis consultation with yourself?

Who would inform a patient who was on the clinical care pathway for suspected heart failure?

REFERRAL

When is a patient referred to secondary care?

Are you involved in the referral process?

What are your thoughts on the referral time to secondary care?

What are the next steps after a patient has been diagnosed with heart failure?

What is your role in the continuing care of a patient diagnosed with heart failure?

VIDEO OF CARDIAC OUTPUT RESPONSE TO STRESS (CORS) TEST

We are proposing a novel test which has been demonstrated to improve suspected heart failure diagnosis. This should therefore improve the number of patient referrals to secondary care

PART 2

NEW CORS TEST

This novel test has been demonstrated to improve suspected heart failure diagnosis. This should therefore improve the number of patient referrals to secondary care.

What are your thoughts about this potential improvement in diagnosis being implemented in the clinical care pathway for heart failure?

Prompt: What is your understanding of the new test?

Was there any information in the demonstration video that was difficult to understand?

Would a patient with a suspected heart failure diagnosis be able to complete this test?

Prompt experience of working with heart failure patients

Could this test be added into a routine consultation for suspected heart failure diagnosis? Or would you expect the patient need to attend a separate appointment to complete the test?

Prompt logistics and timing, space, staff availability.

Do you anticipate any immediate barriers to implementing this test as part of your routine clinical care for a patient with a suspected heart failure diagnosis?

How easy do you think it will be to implement this test in practice?

If this test was to become a routine part of clinical care for a patient suspected with a heart failure diagnosis, who would you expect to deliver it?

Prompt: rational for suggestion

What problems or difficulties do you think you may encounter when using this test?

What problems or difficulties do you think you may encounter with patients performing this test?

What else might you need to deliver this test in practice?

What training would you (or a colleague) need to be able to run this test?

How confident would you be about running this test?

How likely is it that this test will become part of routine clinical care for a patient with a suspected heart failure diagnosis?

Where in the clinical care pathway for suspected heart failure diagnosis would you anticipate this test being implemented?

Prompt: rational for opinion

Where do you think this test would be delivered in your practice?

Do you have any further comments about running this test as part of your routine clinical care for a patient with a suspected heart failure diagnosis?

Interview Schedule v1: Practice Manager (Primary Care)

Project Title: Confirmation of Acceptability of a Novel Cardiac Output Response to Stress (CORS) Test to Improve Diagnosis of Heart Failure in Primary Care

PART 1

CLINICAL CARE PATHWAY

Who would perform these tests in your practice?

How many times is a patient seen before they are referred to secondary care with suspected heart failure diagnosis?

How long is a typical consultation with a patient with a suspected heart failure diagnosis?

REFERRAL

A patient with a moderate to high NT-proBNP reading requires assessment by a specialist and echocardiogram in a heart failure diagnostic clinic in secondary care.

What is your role in the referral process?

What are your thoughts on the referral time to secondary care?

When do you expect to receive the outcome from the referral?

What are the next steps after a patient has been diagnosed with heart failure?

Do the referral times from suspected heart failure diagnosis in primary care to confirmed diagnosis need to be improved?

VIDEO OF CARDIAC OUTPUT RESPONSE TO STRESS (CORS) TEST

We are proposing a novel test which has been demonstrated to improve suspected heart failure diagnosis. This should therefore improve the number of patient referrals to secondary care

PART 2

NEW CORS TEST

This novel test has been demonstrated to improve suspected heart failure diagnosis. This should therefore improve the number of patient referrals to secondary care.

What are your thoughts about this potential improvement in diagnosis being implemented in the clinical care pathway for heart failure?

Prompt: is this test necessary?

What is your understanding of the new test?

Was there any information in the demonstration video that was difficult to understand?

Would you expect commissioners to supply this equipment for each practice?

Prompt: who carries the burden for implementation?

What are your thoughts on the cost of this test per patient?

How easy would it be to implement this test in your practice?

Do you have the staff capacity to run this test as a routine part of clinical care for suspected heart failure diagnosis?

If this test was to become a routine part of clinical care for a patient with a suspected heart failure diagnosis, who would you expect to deliver it?

Prompt: rational for opinion

What problems or difficulties do you think the nominated colleague, e.g practice nurse may encounter when using this test?

What else might you need to deliver this test in practice?

What training would the nominated colleague, i.e. practice nurse need to be able to run this test?

Where do you think this test would be delivered in your practice?

Do you have any further comments about this test becoming integrated into routine clinical care for a patient with a suspected heart failure diagnosis?
