# Supplementary material for: Opportunities and challenges of a novel cardiac output response to stress (CORS) test to enhance diagnosis of heart failure in primary care: qualitative study
Source: BMJ Open. 2019 Apr 14;9(4):e028122. doi: 10.1136/bmjopen-2018-028122 (PMC6500186; doi:10.1136/bmjopen-2018-028122)
Supplement: Author's manuscript [file bmjopen-2018-028122.draft_revisions.pdf]

BMJ Open

BMJ Open is committed to open peer review. As part of this commitment we make the peer review history of every article we publish publicly available.

When an article is published we post the peer reviewers' comments and the authors' responses online. We also post the versions of the paper that were used during peer review. These are the versions that the peer review comments apply to.

The versions of the paper that follow are the versions that were submitted during the peer review process. They are not the versions of record or the final published versions. They should not be cited or distributed as the published version of this manuscript.

BMJ Open is an open access journal and the full, final, typeset and author-corrected version of record of the manuscript is available on our site with no access controls, subscription charges or pay-per-view fees (http://bmjopen.bmj.com).

If you have any questions on BMJ Open's open peer review process please email info.bmjopen@bmj.com

BMJ Open

# Opportunities and challenges of a novel cardiac output response to stress (CORS) test to enhance diagnosis of heart failure in primary care: a qualitative study

| | |
|---|---|
| Journal: | *BMJ Open* |
| Manuscript ID | bmjopen-2018-028122 |
| Article Type: | Research |
| Date Submitted by the Author: | 22-Nov-2018 |
| Complete List of Authors: | Charman, Sarah; Newcastle University, Cardiovascular Research Centre<br>Okwose, Nduka; Newcastle University, Cardiovascular Research Centre<br>Maniatopoulos, Gregory ; Newcastle University, Institute of Health and Society<br>Graziadio, Sara; Newcastle University, NIHR In Vitro Diagnostics Co-operative<br>Metzler, Tamara; Newcastle University, Cardiovascular Research Centre<br>Banks, Helen; Newcastle University, Cardiovascular Research Centre<br>Vale, Luke; Newcastle University, Health Economics Group, Institute of Health and Society<br>MacGowan, Guy; Newcastle University, Institute of Genetic Medicine; Freeman Hospital, Cardiology<br>Seferović, Petar ; University of Belgrade School of Medicine<br>Fuat, Ahmet; Carmel Medical Practice,<br>Deaton, Christi; University of Cambridge, Cambridge Institute of Public Health, School of Clinical Medicine<br>Mant, Jonathan; University of Cambridge, General Practice and Primary Care Research Unit<br>Hobbs, Richard; University of Oxford, Dept of Primary Health Care<br>Jakovljevic, Djordje; Newcastle University, Institute for Ageing and Health |
| Keywords: | Heart failure < CARDIOLOGY, Diagnosis, PRIMARY CARE, QUALITATIVE RESEARCH |
| | |

SCHOLARONE™
Manuscripts

**Opportunities and challenges of a novel cardiac output response to stress (CORS) test to enhance diagnosis of heart failure in primary care: a qualitative study**

Sarah J Charman[1, 2], Nduka C Okwose[1, 2], Gregory Maniatopoulos[3], Sara Graziadio[4], Tamara Metzler[1], Helen Banks[1], Luke Vale[3], Guy A MacGowan[1,2], Petar M Seferovic[5], Ahmet Fuat[6], Christie Deaton[7], Jonathan Mant[7], F.D. Richard Hobbs[8], Djordje G Jakovljevic[1, 2 ,9]

[1] Cardiovascular Research Centre, Institutes of Cellular and Genetic Medicine, Faculty of Medical Sciences, Newcastle University, Newcastle upon Tyne, United Kingdom

[2] Cardiology Department, Freeman Hospital and Royal Victoria Infirmary, Newcastle upon Tyne Hospitals NHS Foundation Trust, Newcastle upon Tyne, United Kingdom

[3] Institute of Health and Society, Newcastle University, United Kingdom

[4] NIHR In Vitro Diagnostics Co-operative, Newcastle upon Tyne Hospitals NHS Foundation Trust , Newcastle upon Tyne, United Kingdom

[5] Cardiology Department, Medical School, University of Belgrade, Clinical Centre Serbia and Serbian Academy of Science and Arts, Serbia

[6] Darlington Memorial Hospital, County Durham and Darlington NHS Foundation Trust & School of Medicine, Pharmacy and Health, Durham University, United Kingdom

[7] Primary Care Unit, Department of Public Health and Primary Care, University of Cambridge, Cambridge, United Kingdom

[8] Nuffield Department of Primary Health Care Sciences, University of Oxford, Oxford, United Kingdom

[9] RCUK Centre for Ageing and Vitality, Newcastle University, Newcastle Upon Tyne, United Kingdom

**Corresponding Author:** Dr Djordje Jakovljevic. Cardiovascular Research Centre, Faculty of Medical Sciences, Newcastle University, and Newcastle upon Tyne Hospitals NHS Foundation Trust, Newcastle upon Tyne, NE2 4HH, United Kingdom. Email: djordje.jakovljevic@newcastle.ac.uk. Tel: +441912088264

For peer review only



For peer review only - http://bmjopen.bmj.com/site/about/guidelines.xhtml

**Strengths and limitations of this study**

- We have recently developed a novel cardiac output response to stress (CORS) test to improve diagnostic accuracy of heart failure in primary care.

- The interviews with 14 health care professionals allowed exploration of the opportunities and challenges for the implementation of the CORS test.

- Study participants were recruited from one geographic region i.e. the North East of England.

For peer review only



For peer review only - http://bmjopen.bmj.com/site/about/guidelines.xhtml

**Abstract**

**Objective:** To determine primary and secondary care healthcare professional's perceptions of the current clinical care diagnostic pathway for heart failure and the potential implementation a novel Cardiac Output Response to Stress (CORS) test developed to improve diagnosis of heart failure.

**Design:** Qualitative study using semi-structured in-depth interviews which were audio recorded and transcribed verbatim. Data from the interviews were analysed thematically using an inductive approach.

**Setting:** Newcastle upon Tyne, UK**.**

**Participants:** Fourteen healthcare professionals (six males, eight females) from primary (general practitioners, nurses, healthcare assistant, practice managers) and secondary care (consultant cardiologists).

**Results:** Six broad themes relating to opportunities and challenges surrounding the implementation of the new diagnostic technology were: 1) clinical need in the current pathway, 2) evidence requirement to inform the potential role of the test 3) patient usability and acceptability 4) costs and resources 5) capacity (and environment) 6) capability.

**Conclusion:** There is high clinical demand for access to an objective cardiac function test in primary care to facilitate diagnostic and referral accuracy of heart failure. Further evidence of the clinical- and cost-effectiveness of the CORS test is required prior to implementation.

**Key words:** diagnosis, heart failure, primary care, qualitative research.



For peer review only - http://bmjopen.bmj.com/site/about/guidelines.xhtml

**Introduction**

Heart failure is a complex clinical syndrome resulting from impaired heart function (1, 2). It is characterised by debilitating symptoms for the patient, including breathlessness, oedema and fatigue and often co-exists with other comorbidities (3). Signs and symptoms are non-specific and therefore difficult for primary care physicians to produce an accurate diagnosis and referral (4). Increasing ageing population and improved outcomes in acute coronary syndrome have led to a growing incidence and prevalence of heart failure. Heart failure (HF) is recognised globally as a challenging public health burden (5).

Early diagnosis of HF is crucial to improve survival, reduce hospitalisation and costs (1, 6). Primary care represents the first step in the clinical care pathway for patients presenting with symptoms of HF and General Practitioners (GPs) play a crucial role in early identification (7, 8). The diagnostic tools currently available to GPs include electrocardiography (which is insufficiently sensitive to detect HF (9), and serum natriuretic peptides, accurate as a 'rule out' test to exclude HF but can lead to a significant number of false positives due to its low specificity (10-12). As a result, >65% of suspected HF patients referred from primary to secondary care do not have diagnosis confirmed following echocardiography and specialist review (4, 9, 13, 14). This creates undue worry for the patient with the average waiting time for investigation being 67 days (15), delays in care of patients who have HF, an unnecessary burden and costs to secondary care (1, 2, 16). There is a role for an additional diagnostic tool to help GPs identify impaired heart function and improve diagnosis and referral accuracy for HF.

We have recently developed and confirmed acceptable reproducibility of a novel, easy-to-use, non-invasive cardiac output response to stress (CORS) test (Figure 1) for evaluation of cardiac function in primary care (17). The CORS test has the potential to be utilised by healthcare professionals to improve



For peer review only - http://bmjopen.bmj.com/site/about/guidelines.xhtml

diagnostic accuracy for suspected heart failure and provide an objective measure of cardiac function as a ‘rule in’ test. Prior to conducting a large trial on further effectiveness of the CORS test, it was important to understand how primary and secondary healthcare professionals perceive this new diagnostic test. The objective of this study was to determine the current clinical care pathway for suspected HF adopted by healthcare professionals, and to identify the potential opportunities and challenges to implementation of the CORS test in the clinical practice.

## Methods

### Study design, setting and participants

Qualitative study using semi-structured in-depth interviews which were audio recorded and transcribed verbatim. Data from the interviews were analysed thematically using an inductive approach. Semi-structured in-depth interviews (14 in total; Table 1) were conducted between December 2017 and April 2018 in the North East of England. Participants were purposively selected according to their role and involvement in the management of patients with suspected or diagnosed heart failure in both primary and secondary care. Potential interviewees were sent an email invitation which briefly outlined the aims of the study. Those agreeing to participate were invited to recommend additional candidates for interview. Data were collected at the workplace of study participants. The study protocol (number 15/NE/0190) was approved by the National Health Service, National Research Authority (North East – Tyne & Wear South Research Ethics Committee). All interviews were digitally recorded, anonymized and transcribed in full. One Research Associate (SJC, PhD-female) conducted the fieldwork. The researcher completed PhD programme based on knowledge and application of mixed methods including qualitative methods. The researcher did not establish relationship with the study participants prior study commencement.



For peer review only - http://bmjopen.bmj.com/site/about/guidelines.xhtml

Interviews were typically around 60 min in length and conducted on an individual, face-to-face basis. The final sample is described in Table 1, and included: four GPs, one practice nurse, two practice managers, one healthcare assistant, three heart failure specialist community nurses, three consultant cardiologists with a specialist interest in heart failure. Firstly, participants were asked questions on the current clinical care pathway for heart failure. Then, a video demonstration of a patient completing the Cardiac Output Response to Stress (CORS) test was shown with an explanation of each phase of the test provided by the interviewer (duration: 02:41 minutes). Finally, questions were asked about the CORS test and potential barriers and facilitators to its implementation.

**The CORS test**

The CORS test (Figure 1) has been previously described (17) and consists of four phases: 1) rest, where the patient lies in the supine position for 3 minutes; 2) challenge, where the patient remains in the standing position for 3 minutes; 3 and 4) stress exercise, where a patient completes a step test with continuous haemodynamic measurements (i.e. cardiac output and cardiac index) using non-invasive, electrical signal processing technology.

**Data analysis**

Data from the interviews was analysed thematically using an inductive approach (18). All interviews were transcribed verbatim. Transcripts were grouped by healthcare professional role (GP, practice nurse, healthcare assistant, practice manager, consultant cardiologist) and subdivided in to primary and secondary care organisational role. For the purposes of inter-coder reliability, two (SJC and NCO) researchers analysed the data independently. These were later discussed and approved by the research team.



For peer review only - http://bmjopen.bmj.com/site/about/guidelines.xhtml

For peer review only

## Results

Analysis of the data generated six broad themes relating to opportunities and challenges surrounding the implementation of the new diagnostic technology: 1) clinical need in the current pathway, 2) evidence requirement to inform the potential role of the test 3) patient usability and acceptability 4) costs and resources 5) capacity (and environment) 6) capability.

### Summary of the current clinical care pathway for suspected heart failure

Figure 2 provides a flowchart that describes the current clinical care pathway for chronic heart failure, incorporating NICE guidance (2, 3), and the care pathway descriptions provided during the interviews.

### Theme 1: Clinical need in the current pathway

Participants suggested that HF was difficult to diagnose with the patient often presenting with more than one complication or comorbidity, usually when disease exacerbates.

*“If somebody came in who had had a previous MI and said, "My ankles have swelled up. I can't lie flat in bed at night," and they had nice crackles at the bottom of their lungs, you would probably be right there and then saying, "I think this might be related to your heart," and so on. But sometimes it's much less obvious. It's just somebody presenting with breathlessness and it could be anything from heart failure to lung cancer, COPD. I mean I'm thinking of a particular patient who did end up having, in the end, quite severe HF but it wasn't obvious at all”.* (GP, Practice 3).

It was acknowledged by secondary care participants that in primary care HF is difficult to diagnose based on the limited diagnostics available in primary care including natriuretic peptides, which can be elevated due to increasing age or other comorbidities.



For peer review only - http://bmjopen.bmj.com/site/about/guidelines.xhtml

*"I think you have to see quite a high proportion of people who do not have HF to avoid missing it. I also think it's quite a hard diagnosis in primary care because the blood tests are a very blunt tool. They're great for rule out but they do not rule in. Because the levels go up with age, they go up with multiple comorbidities. Many patients who are thought to have HF, actually will have a raised level, even if they turn out not to have HF".* (Consultant Cardiologist 2, Hospital site).

GPs did not report a problem with either the referral waiting timeframe or receiving the outcome of the referral. GPs suggested that patients are often seen by a number of different GPs, so there is often a delay in a referral or a lack of confidence to make a decision resulting in poor referral. One of the Consultant Cardiologists was sympathetic with these concerns and acknowledged that the point of referral was different between practices and noted staff "*experience as well as knowledge and skill and confidence"* play an important role in this process. A GP suggested that the process takes longer due to the complexity of the condition and the lack of priority HF is given in comparison to other conditions.

Although the referral waiting times for patients with suspected HF were not an area of particular concern for secondary care clinicians, identifying the appropriate patient was crucial and understandably problematic. Participants acknowledged that they often receive many inaccurate referrals through the elective admission route which led to wasted consultant time.

*"That's the issue. We're seeing too many patients who we don't really need to see. If they showed me an echo just to report on an ECG I would say, "I don't need to see them." But the way it's set up, it's very inefficient".* (Consultant Cardiologist 1, Hospital site).



For peer review only - http://bmjopen.bmj.com/site/about/guidelines.xhtml

**Theme 2: Evidence requirement to inform the potential role of the test**

Participants highlighted the role of evidence in implementing a pathway for the new diagnostic technology.

*"Yes. I would want to know what's the evidence for it, how clear cut is the interpretation and actually, if this is just going to be another pre-test that is done before the people go, then what exactly is the role of the clinic".* (GP, Practice 3).

Although improvements in both referral accuracy and patient care were considered to be important for secondary care clinicians, it was difficult to determine where the CORS test would fit in the current clinical care pathway without knowing its efficacy. Secondary care clinicians reported that the implementation of the new diagnostic would be less useful in secondary care with the patient already receiving an echocardiogram.

*"If it's proven to be effective in terms of being able to distinguish between possible HF or refuted diagnosis of HF, then I think it's useful. In terms of where it would fit into a pathway? Then it would appear to be that it would be at the start of the pathway before referring to secondary care."* (Consultant Cardiologist 3, Hospital site).

Successful implementation of the CORS test would be dependent upon confirmation of clinical and cost effectiveness and convincing both GP partners and nurses regarding the clinical need for it and the value of completing the test and providing good patient care.

*"You need more evidence, is the key bit. So it needs the evidence behind it. I think you might need assessment through NICE to see what they think it is as an additional test".* (Consultant Cardiologist 2, Hospital site).



For peer review only - http://bmjopen.bmj.com/site/about/guidelines.xhtml

**Theme 3: Patient usability and acceptability**

A number of potential patient characteristics that could lead to patient exclusion were identified i.e. mobility, possible difficulties lying down, need of a balance aid, suggesting that not every patient would be suitable. Exercise was identified as potentially challenging. The number of patients who could potentially benefit from this technology could be increased if the duration of Phase 3 and 4 could be reduced.

*"We have some patients with established HF for example, you don't even recommend they weigh themselves every day because of the safety of stepping on to the scales. So I think there needs that recognition that some patients may not be suitable for it."* (Consultant Cardiologist 2, Hospital site).

Identifying appropriate patients may be difficult due to mobility, frailty and balance issues. Other participants suggested possibility of using a set of parallel bars. GPs provided the test uptake estimates 50-80% for completing the CORS test. The practice nurse was far more optimistic with an estimated 80% uptake.

**Theme 4: Cost and resources**

Overall, GPs were very positive about the potential role of the CORS test in primary care to aid diagnosis of HF and provided reasons regarding the large numbers of undiagnosed cases of the condition.

*"Personally, I think this would be a great idea, because I think it would be, if it's more accurate, it would actually certainly reduce wastage in terms of referrals. And it would allow us to make the diagnosis much earlier and manage the patient based on that, really…. Well, you're talking to someone who believes that 70 per cent of HF is undiagnosed. And I don't know what the figure is, but most HF, I believe, is currently diagnosed in the AU (emergency admission unit)….So, you know, you're talking to*



For peer review only - http://bmjopen.bmj.com/site/about/guidelines.xhtml

*someone who thinks that anything we can do which actually makes it easier to diagnose HF in primary care is a good thing".* (GP, Practice 2).

However, some were sceptical about the implementation of the CORS test and suggested that they would need greater convincing about the time needed, skills expected to deliver, cost and administration of the test.

An additional appointment would be required to implement the CORS in primary care. A healthcare assistant suggested an integration of the CORS test during the current ECG and biomarker testing appointment. The practice managers acknowledged that an additional appointment will incur an additional cost to the practice but also the availability of appointments would be a potential issue.

*"Because, it wouldn't replace existing appointments, additional appointments. That in itself adds a cost to the practice, that we would have to look at….That is the overall driver. You know, that assessment, obviously, has to be made at a partner and business level. The second thing, that always drives us, is cost. The third thing is impact on availability of appointments. Of course, it's availability of appointments is about the only thing as a practice we are actually measured on".* (Practice Manager, Practice 1).

Practice managers acknowledged that finances do not always support the difference between additional work and capacity to increase the workforce. This was supported from a GP perspective who emphasised that practices are told to be realistic on what they can and cannot deliver.

*"There's increasingly a feeling that not all practices can deliver all services at all times. However, it's more reasonable to work to the premise that all the patients of all the practices should have access to all the tests they need. But maybe just not in their own building".* (GP, Practice 2).



For peer review only - http://bmjopen.bmj.com/site/about/guidelines.xhtml

In addressing these challenges it was suggested that a potential setting for delivery of the CORS could be at the community setting such as community diagnostic centres or outreach diagnostic facility.

The cost of the equipment was also acknowledged as a potential barrier to the implementation. One approach to commissioning a new pathway is to incentivise primary care through negotiation between the primary care contractors and their Clinical Commissioning Groups.

*"I wouldn't expect them to supply it. I would expect them to fund the equipment initially. They would normally pass on subsequent maintenance costs to us. I would also expect them to commission this as an enhanced service. So, that we can recoup the manpower and equipment costs of doing it".* (Practice Manager, Practice 1).

**Theme 5: Capacity (and environment)**

A variety of responses were given regarding the designated professional to deliver the CORS test i.e. GPs and nurses suggested either a practice nurse or healthcare assistant. The healthcare assistant agreed that they could deliver this test given their experience of performing ECGs but would need practice and training. Practice managers suggested that nurses could potentially be the best person to deliver it.

For Consultant Cardiologists, a qualified nurse would be the best person to administer the test in primary care but others suggested local GPs practice should guide the selection of the most appropriate person for the delivery of the CORS test *"you need somebody who can say, "Keep going," but you also need someone to say, "No, stop. You look absolutely awful, stop.""* (Consultant Cardiologist 2, Hospital site).



For peer review only - http://bmjopen.bmj.com/site/about/guidelines.xhtml

Practice managers identified the space for storage of the equipment and as a potential issue. Differences in the building capacity were acknowledged between practices by the practice managers. A practice nurse suggested that the receptionists would need to be aware what room the equipment was in and follow similar booking process as for the ECG.

**Theme 6: Capability**

Training would be essential and reported as a necessity by all healthcare professionals in primary care. It was suggested that not only the professional delivering the test would need to be trained, but also the professional interpreting the results. For example, a healthcare assistant would deliver the test but a GP would interpret it, in much the same process as currently occurs for ECGs. There was no preference between individual or group training but the healthcare assistant suggested group training would be less time consuming.

The complexity of the technology interface was also a concern for practice managers, in particular in terms of how often a nurse would be using the equipment to be confident with the delivery and outcomes. Participants expressed concern in relation to some of the technical issues i.e. data integration needed to ensure results are inputted on to patient records for interpretation purposes.

*"From a management perspective, my real concern is also how complicated the technology is. How easy it is for a practice nurse, who may not be using it day in day out to operate it?….. You've done this test…How do you then get it to the doctor?".* (Practice Manager, Practice 1).

The safety component for delivering an exercise test in primary care was raised.

All these practical barriers require solutions prior to implementation of the CORS test.



For peer review only - http://bmjopen.bmj.com/site/about/guidelines.xhtml

For peer review only

**Discussion**

Our findings have clearly identified a number of opportunities and challenges that would need to be addressed during the next phases in refinement of the CORS test. The study captured primary and secondary care healthcare professional's perceptions for the implementation of a new diagnostic test. There is a clinical need to improve diagnostic tools available in primary care, in particular to rule in HF using a high specificity test. This would reduce unnecessary visits to secondary care for those without HF and reduce inaccurate referrals.

Major finding suggests that not every patient would be suitable for to CORS test. This leads two possible scenarios: 1) Not changing the characteristics of the CORS test, and restricting the patient inclusion criteria for the next evaluation phase to patients with no major mobility issues, or 2) Adapting the CORS test by shortening the phase 3 and 4, considering alternative positions (sitting or passive leg raise) using supporting aids during the exercise phase. The CORS test will be adopted accordingly and diagnostic accuracy and cost-effectiveness will be evaluated. After collection of the necessary evidence, an application to the NICE's Diagnostic Assessment Programme will be attempted to increase the possibilities of widespread implementation and adoption in primary care.

Secondary care participants questioned test ability to differentiate between HF reduced vs HF preserved ejection fraction. Patients with HF reduced ejection fraction demonstrate 25-30% lower resting cardiac and stroke volume index by 25-37% compared to matched controls (19-23). HF with preserved ejection fraction demonstrate both reduced and normal resting cardiac index (20, 21, 24, 25), but significantly diminished cardiac output response to stress (19, 20, 24).



For peer review only - http://bmjopen.bmj.com/site/about/guidelines.xhtml

**Strengths and limitations**

This is the first study to explore the opportunities and challenges for the implementation of the CORS test. The perspective of primary and secondary healthcare professionals was considered, which ensured a more reflexive analysis of data (26). This approach allowed identification of suboptimal communication between primary and secondary care in the current HF clinical care pathway, which delays diagnosis and results in inaccurate referrals and wasted consultant time.

Given that participants were selected only from a region of the North East of England and in small numbers meant our findings may have limited generalisation. Data were analysed thematically using an inductive approach, which led to saturation of themes at the analysis stage (27). However, this was the first iteration of the analysis of opportunities and challenges for the CORS test to inform further refinement of the CORS test.

**Comparison with existing literature**

HF is a growing burden in the UK with new diagnoses increasing by 12% between 2002 and 2014 due to ageing population (28). The current clinical care pathway (2) and selected diagnostic tests highlighted by healthcare professionals suggest an accurate diagnosis is difficult in primary care highlighting our first theme – the clinical need in the current pathway. GPs are often blinded by more than one comorbidity leading to misdiagnosis (29). The CORE Needs Assessment Survey highlighted that clinicians are only considering half the symptoms and signs for HF, which addresses why the diagnosis continues to be difficult, highlighting the need of awareness of other co-existing signs and symptoms (30).

Data suggest that patients with suspected HF are not always seen by the same GP and this led to delays or a lack of confidence to make appropriate decision. Lack of knowledge about HF from GPs can result in 'mismanaged patients' (31) and differing opinions between GPs within a practice is not



For peer review only - http://bmjopen.bmj.com/site/about/guidelines.xhtml

uncommon during the clinical diagnosis for heart failure (32), which both result in inconsistencies in patient care. The potential introduction of a new diagnostic test such as the CORS could provide GPs with an objective measure of heart function (17), which has the potential to lead to less discrepancies in outcomes and more informed decision making.

Echocardiography performed and interpreted in secondary care is non-invasive and cost effective imaging technique to aid diagnosis of HF (1-3). Khunti et al (2002) (32) reported that very few GPs sent patients for echocardiography to confirm diagnosis, but the participants in the present study were aware of the benefits of this test as part of the referral process to confirm or refute a diagnosis of HF. Howlett et al (2018) (30) however reported that primary care clinicians and nurses had low confidence in performing and interpreting echocardiography as part of the diagnostic testing for HF.

**Implications for research and/or practice**

Healthcare professionals were able to identify opportunities and challenges to the implementation of the CORS test. It requires adequate funding to be implemented and support from both clinical champions and commissioners. The capacity within primary care such as cost, space and time need to be accounted for and these challenges may be addressed by the positioning of the CORS test within a community setting (i.e. community diagnostic centres or outreach diagnostic facility). This would potentially reduce the burden on the practice to deliver the test. However, it is reasonable to suggest that potential benefits may outweigh potential challenges and thus lead to implementation of the CORS test in primary care.

Health care professional training is needed and expected with any new diagnostic test. Data suggest either a practice nurse or healthcare assistant would be suitable to deliver the CORS test. Our previous study demonstrated acceptable reproducibility of the CORS test (17). The present study highlights potential issues to the CORS test implementation and ongoing research aims to address these



For peer review only - http://bmjopen.bmj.com/site/about/guidelines.xhtml

challenges. We plan to make the CORS test accessible for the majority of patients entering the clinical care pathway. This will be achieved by reducing duration of the exercise component and adding a passive leg raise haemodynamic challenge during the resting phase of the CORS test, which shows preliminary accuracy to identify cardiac dysfunction.

**Contributors:** *Study concept and design:* DGJ, SJC; *Acquisition of data:* SJC, NCO; *Data analysis and interpretation of data:* SJC, NCO; *Drafting of the manuscript:* SJC, GM, SG, NCO, DGJ; *Critical revision of the manuscript for important intellectual content:* TM, HB, LV, GAM, PMS, AF, CD, JM, FDRH. *Administrative, technical, or material support:* SJC, NCO; *Study Supervision:* DGJ. All authors critically reviewed the manuscript and approved the final version. DGJ acts as the guarantor and takes responsibility for the content of the manuscript, including the data and analysis.

**Funding**

This study was funded by the UK Medical Research Council Confidence in Concept Scheme grant to D.G.J. (grant no. BH161161). S.J.C. and N.O. are supported by the European Horizon 2020 research and innovation programme under grant agreement No 777204. S.G is supported by the NIHR MEDTEch In Vitro Diagnostics. D.G.J. is supported by the UK Research Councils' Newcastle Centre for Ageing and Vitality (grant no. L016354).The views expressed are those of the authors and not necessarily those of the Medical Research Council. The funders had no role in study design or in data collection, analysis, or interpretation.



For peer review only - http://bmjopen.bmj.com/site/about/guidelines.xhtml

**Ethical approval**

The study protocol (number 15/NE/0190) was approved by the National Health Service, National Research Authority (North East – Tyne & Wear South Research Ethics Committee). All procedures performed in the study were in accordance with the Declaration of Helsinki.

**Competing interests**

The authors have no relationships or activities that could appear to have influenced the submitted work.

**Acknowledgements** We would like to thank all healthcare professionals who gave their time to being interviewed and participating in this study.

**Data sharing:** the relevant anonymised patient level data are available on reasonable request from the authors.

**Transparency:** The lead author (DGJ) affirms that the manuscript is an honest, accurate, and transparent account of the study being reported; that no important aspects of the study have been omitted; and that any discrepancies from the study as planned have been explained. The corresponding author had full access to all the study data and final responsibility for publication submission.

**Licence:** The Corresponding Author has the right to grant on behalf of all authors and does grant on behalf of all authors, a worldwide licence to the Publishers and its licensees in perpetuity, in all forms, formats and media (whether known now or created in the future), to i) publish, reproduce, distribute, display and store the Contribution, ii) translate the Contribution into other languages, create adaptations, reprints, include within collections and create summaries, extracts and/or, abstracts of

the Contribution and convert or allow conversion into any format including without limitation audio, iii) create any other derivative work(s) based in whole or part on the on the Contribution, iv) to exploit all subsidiary rights to exploit all subsidiary rights that currently exist or as may exist in the future in the Contribution, v) the inclusion of electronic links from the Contribution to third party material where-ever it may be located; and, vi) licence any third party to do any or all of the above.

For peer review only

**References**

1. Ponikowski P, Voors AA, Anker SD, et al. 2016 ESC Guidelines for the diagnosis and treatment of acute and chronic heart failure: The Task Force for the diagnosis and treatment of acute and chronic heart failure of the European Society of Cardiology (ESC). Developed with the special contribution of the Heart Failure Association (HFA) of the ESC. Eur J Heart Fail. 2016;18(8):891-975.

2. National Institute for Health and Care Excellence. Chronic heart failure in adults: Diagnosis and management. In: NG106, editor. London: Royal College of Physicians; 2018.

3. National Institute for Health and Clinical Excellence. Chronic Heart Failure in Adults: Management. In: [CG108] Cg, editor. London: National Clinical Guideline Centre; 2010.

4. Fuat A, Hungin AP, Murphy JJ. Barriers to accurate diagnosis and effective management of heart failure in primary care: qualitative study. BMJ. 2003;326(7382):196.

5. McMurray JJV, Stewart S. The burden of heart failure. Eur Heart J Suppl. 2002;4(suppl_D):D50-D8.

6. Hobbs FD, Kenkre JE, Roalfe AK, et al. Impact of heart failure and left ventricular systolic dysfunction on quality of life: a cross-sectional study comparing common chronic cardiac and medical disorders and a representative adult population. Eur Heart J. 2002;23(23):1867-76.

7. Taylor CJ, Rutten FH, Brouwer JR, Hobbs FR. Practical guidance on heart failure diagnosis and management in primary care: recent EPCCS recommendations. Br J Gen Pract. 2017;67(660):326-7.

8. Roalfe AK, Mant J, Doust JA, et al. Development and initial validation of a simple clinical decision tool to predict the presence of heart failure in primary care: the MICE (Male, Infarction, Crepitations, Edema) rule. Eur J Heart Fail. 2012;14(9):1000-8.

9. Khunti K, Squire I, Abrams KR, Sutton AJ. Accuracy of a 12-lead electrocardiogram in screening patients with suspected heart failure for open access echocardiography: a systematic review and meta-analysis. Eur J Heart Fail. 2004;6(5):571-6.

10. Taylor CJ, Roalfe AK, Iles R, et al. Primary care REFerral for EchocaRdiogram (REFER) in heart failure: a diagnostic accuracy study. Br J Gen Pract. 2017;67(655):e94-e102.



For peer review only - http://bmjopen.bmj.com/site/about/guidelines.xhtml

11. Roberts E, Ludman AJ, Dworzynski K, et al. The diagnostic accuracy of the natriuretic peptides in heart failure: systematic review and diagnostic meta-analysis in the acute care setting. BMJ. 2015;350:h910.

12. Hobbs FD, Doust J, Mant J, Cowie MR. Heart failure: Diagnosis of heart failure in primary care. Heart. 2010;96(21):1773-7.

13. Kelder JC, Cramer MJ, van Wijngaarden J, et al. The diagnostic value of physical examination and additional testing in primary care patients with suspected heart failure. Circulation. 2011;124(25):2865-73.

14. Fonseca C. Diagnosis of heart failure in primary care. Heart Fail Rev. 2006;11(2):95-107.

15. Fazal IA, Bhagra SK, Bailey KM, et al. Impact of using different guideline recommended serum natriuretic peptide thresholds on the diagnosis and referral rates of a diagnostic heart failure clinic. Int J Clin Pract. 2015;69(11):1349-56.

16. Benjamin EJ, Virani SS, Callaway CW, et al. Heart Disease and Stroke Statistics-2018 Update: A Report From the American Heart Association. Circulation. 2018;137(12):e67-e492.

17. Charman SJ, Okwose NC, Stefanetti RJ, et al. A novel cardiac output response to stress test developed to improve diagnosis and monitoring of heart failure in primary care. ESC Heart Fail. 2018;5(4):703-12.

18. Braun V, Clarke V. Using thematic analysis in psychology. Qual Res Psychol. 2006;3(2):77-101.

19. Carlsson M, Andersson R, Bloch KM, et al. Cardiac output and cardiac index measured with cardiovascular magnetic resonance in healthy subjects, elite athletes and patients with congestive heart failure. J Cardiovasc Magn Reson. 2012;14:51.

20. Dhakal BP, Malhotra R, Murphy RM, et al. Mechanisms of exercise intolerance in heart failure with preserved ejection fraction: the role of abnormal peripheral oxygen extraction. Circ Heart Fail. 2015;8(2):286-94.

21. Farr MJ, Lang CC, Lamanca JJ, et al. Cardiopulmonary exercise variables in diastolic versus systolic heart failure. Am J Cardiol. 2008;102(2):203-6.



For peer review only - http://bmjopen.bmj.com/site/about/guidelines.xhtml

22. Hudsmith LE, Petersen SE, Francis JM, et al. Normal human left and right ventricular and left atrial dimensions using steady state free precession magnetic resonance imaging. J Cardiovasc Magn Reson. 2005;7(5):775-82.

23. Shelton RJ, Ingle L, Rigby AS, et al. Cardiac output does not limit submaximal exercise capacity in patients with chronic heart failure. Eur J Heart Fail. 2010;12(9):983-9.

24. Zile MR, Kjellstrom B, Bennett T, et al. Effects of exercise on left ventricular systolic and diastolic properties in patients with heart failure and a preserved ejection fraction versus heart failure and a reduced ejection fraction. Circ Heart Fail. 2013;6(3):508-16.

25. Abudiab MM, Redfield MM, Melenovsky V, et al. Cardiac output response to exercise in relation to metabolic demand in heart failure with preserved ejection fraction. Eur J Heart Fail. 2013;15(7):776-85.

26. Mays N, Pope C. Qualitative research in health care. Assessing quality in qualitative research. BMJ. 2000;320(7226):50-2.

27. Saunders B, Sim J, Kingstone T, et al. Saturation in qualitative research: exploring its conceptualization and operationalization. Qual Quant. 2018;52(4):1893-907.

28. Conrad N, Judge A, Tran J, et al. Temporal trends and patterns in heart failure incidence: a population-based study of 4 million individuals. Lancet. 2018;391(10120):572-80.

29. Smeets M, Van Roy S, Aertgeerts B, et al. Improving care for heart failure patients in primary care, GPs' perceptions: a qualitative evidence synthesis. BMJ Open. 2016;6(11).

30. Howlett J, Comin-Colet J, Dickstein K, et al. Clinical practices and attitudes regarding the diagnosis and management of heart failure: findings from the CORE Needs Assessment Survey. ESC Heart Fail. 2018;5(1):172-83.

31. Simmonds R, Glogowska M, McLachlan S, et al. Unplanned admissions and the organisational management of heart failure: a multicentre ethnographic, qualitative study. BMJ Open. 2015;5(10).

For peer review only

32. Khunti K, Hearnshaw H, Baker R, Grimshaw G. Heart failure in primary care: qualitative study of current management and perceived obstacles to evidence-based diagnosis and management by general practitioners. Eur J Heart Fail. 2002;4(6):771-7.

For peer review only



For peer review only - http://bmjopen.bmj.com/site/about/guidelines.xhtml

**List of Tables**

**Table 1:** List of interviewees.

**List of Figures**

**Figure 1:** Cardiac output response to stress test. The cardiac output response to stress test consists of three phases: rest, challenge, and stress exercise. Each phase lasts for 3 min, and stress phase integrates additional 3 min to increase intensity and metabolic demand (from 10 to 15 steps per minute).

**Figure 2:** Current clinical pathway for suspected heart failure diagnosis described by healthcare professionals in this study and adopted from the guidelines from the National Institute for Health and Care Excellence (NICE) for Chronic Heart Failure (2, 3).

For peer review only



For peer review only - http://bmjopen.bmj.com/site/about/guidelines.xhtml

**Table 1.** List of interviewees

| Organisation | No of interviews | Interviewee | Practice list size |
|---|---|---|---|
| Practice 1 | 2 | GP<br>Practice Manager | 5512 |
| Practice 2 | 3 | GP<br>Practice Nurse<br>Practice Manager | 5140 |
| Practice 3 | 1 | GP | 6491 |
| Practice 4 | 2 | GP<br>Healthcare Assistant | 11091 |
| Community setting | 3 | Community Heart Failure Nurse | |
| Hospital site | 3 | Consultant Cardiologist | |
| Total | 14 | | |

For peer review only

**Figure 1**

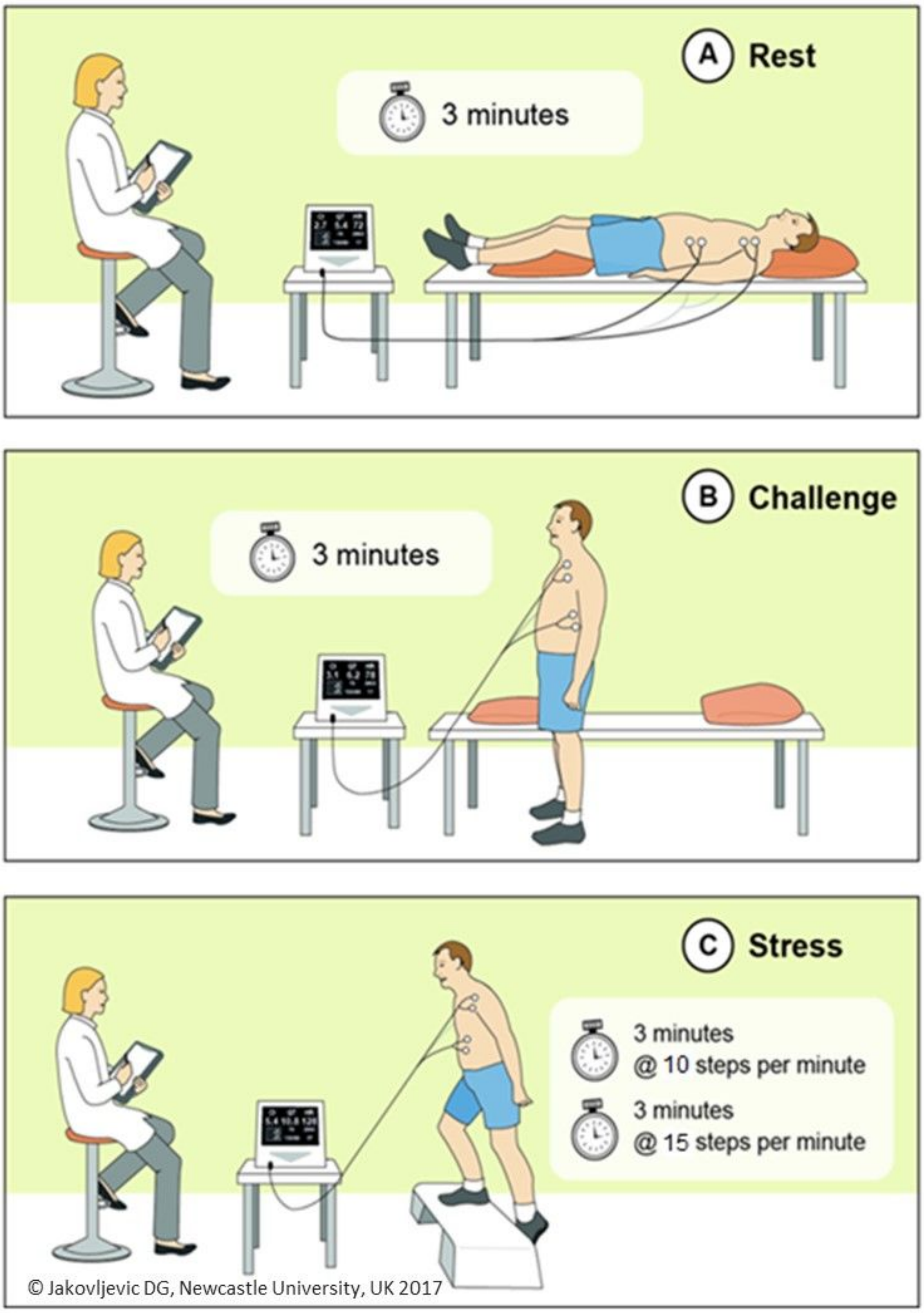
A Rest
3 minutes
B Challenge
3 minutes
C Stress
3 minutes
@ 10 steps per minute
3 minutes
@ 15 steps per minute
© Jakovljevic DG, Newcastle University, UK 2017

Figure 2

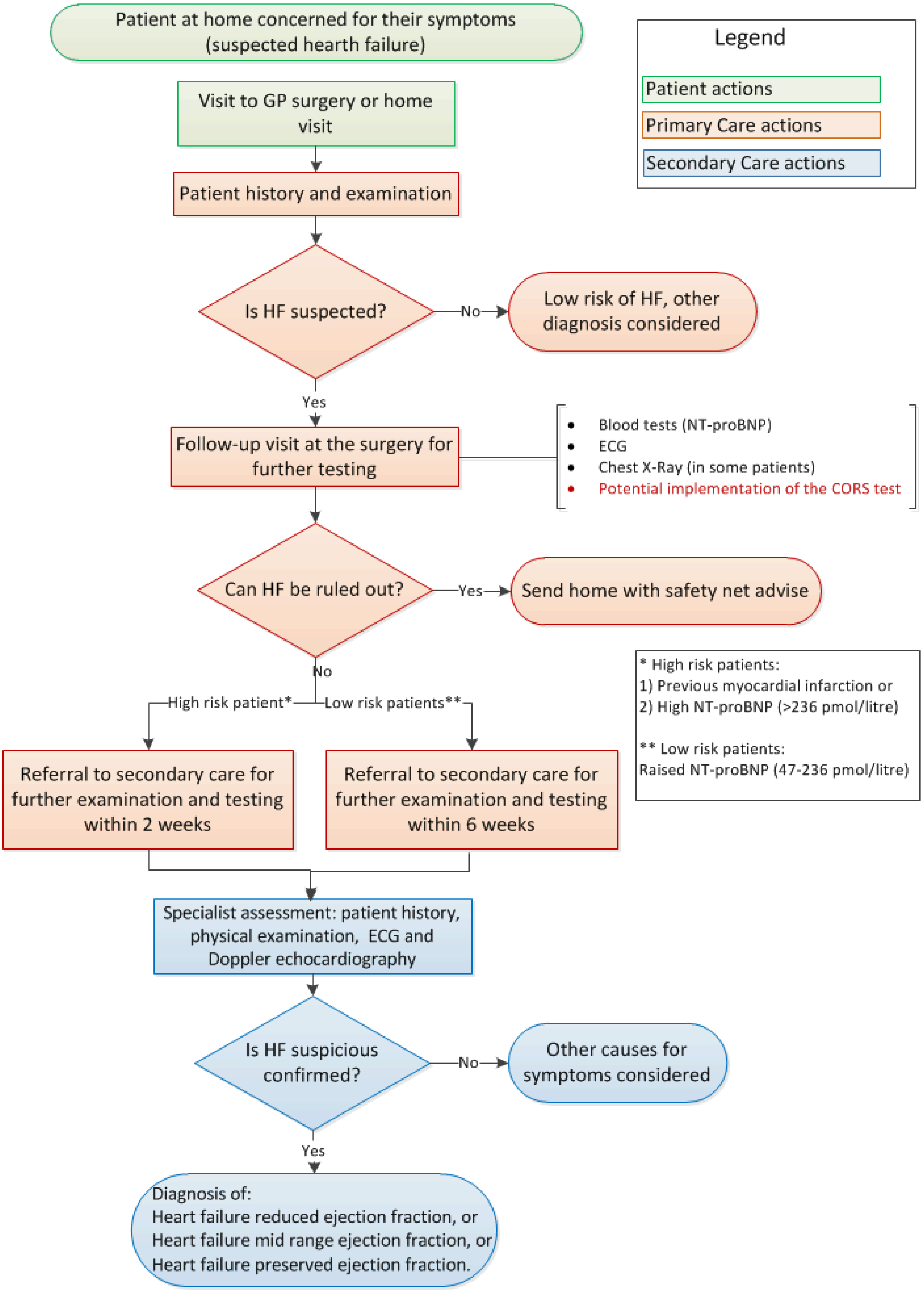
Patient at home concerned for their symptoms (suspected hearth failure)
Visit to GP surgery or home visit
Patient history and examination
Is HF suspected?
No
Low risk of HF, other diagnosis considered
Yes
Follow-up visit at the surgery for further testing
Blood tests (NT-proBNP)
ECG
Chest X-Ray (in some patients)
Potential implementation of the CORS test
Can HF be ruled out?
Yes
Send home with safety net advise
No
High risk patient*
Low risk patients**
Referral to secondary care for further examination and testing within 2 weeks
Referral to secondary care for further examination and testing within 6 weeks
* High risk patients:
1) Previous myocardial infarction or
2) High NT-proBNP (>236 pmol/litre)
** Low risk patients:
Raised NT-proBNP (47-236 pmol/litre)
Specialist assessment: patient history, physical examination, ECG and Doppler echocardiography
Is HF suspicious confirmed?
No
Other causes for symptoms considered
Yes
Diagnosis of:
Heart failure reduced ejection fraction, or
Heart failure mid range ejection fraction, or
Heart failure preserved ejection fraction.
Legend
Patient actions
Primary Care actions
Secondary Care actions

# COREQ (COnsolidated criteria for REporting Qualitative research) Checklist

A checklist of items that should be included in reports of qualitative research. You must report the page number in your manuscript where you consider each of the items listed in this checklist. If you have not included this information, either revise your manuscript accordingly before submitting or note N/A.

| Topic | Item No. | Guide Questions/Description | Reported on Page No. |
|---|---|---|---|
| **Domain 1: Research team and reflexivity** | | | |
| *Personal characteristics* | | | |
| Interviewer/facilitator | 1 | Which author/s conducted the interview or focus group? | 5 |
| Credentials | 2 | What were the researcher's credentials? E.g. PhD, MD | 5 |
| Occupation | 3 | What was their occupation at the time of the study? | 5 |
| Gender | 4 | Was the researcher male or female? | 5 |
| Experience and training | 5 | What experience or training did the researcher have? | 5 |
| *Relationship with participants* | | | |
| Relationship established | 6 | Was a relationship established prior to study commencement? | 5 |
| Participant knowledge of the interviewer | 7 | What did the participants know about the researcher? e.g. personal goals, reasons for doing the research | n/a |
| Interviewer characteristics | 8 | What characteristics were reported about the inter viewer/facilitator? e.g. Bias, assumptions, reasons and interests in the research topic | n/a |
| **Domain 2: Study design** | | | |
| *Theoretical framework* | | | |
| Methodological orientation and Theory | 9 | What methodological orientation was stated to underpin the study? e.g. grounded theory, discourse analysis, ethnography, phenomenology, content analysis | 5,6 |
| *Participant selection* | | | |
| Sampling | 10 | How were participants selected? e.g. purposive, convenience, consecutive, snowball | 5 |
| Method of approach | 11 | How were participants approached? e.g. face-to-face, telephone, mail, email | 5 |
| Sample size | 12 | How many participants were in the study? | 14 |
| Non-participation | 13 | How many people refused to participate or dropped out? Reasons? | n/a |
| *Setting* | | | |
| Setting of data collection | 14 | Where was the data collected? e.g. home, clinic, workplace | 5 |
| Presence of non-participants | 15 | Was anyone else present besides the participants and researchers? | no |
| Description of sample | 16 | What are the important characteristics of the sample? e.g. demographic data, date | n/a |
| *Data collection* | | | |
| Interview guide | 17 | Were questions, prompts, guides provided by the authors? Was it pilot tested? | 6 |
| Repeat interviews | 18 | Were repeat inter views carried out? If yes, how many? | no |
| Audio/visual recording | 19 | Did the research use audio or visual recording to collect the data? | 5 |
| Field notes | 20 | Were field notes made during and/or after the inter view or focus group? | No |
| Duration | 21 | What was the duration of the inter views or focus group? | 6 |
| Data saturation | 22 | Was data saturation discussed? | n/a |
| Transcripts returned | 23 | Were transcripts returned to participants for comment and/or | no |

For peer review only

| Topic | Item No. | Guide Questions/Description | Reported on Page No. |
|---|---|---|---|
| | | correction? | |
| **Domain 3: analysis and findings** | | | |
| *Data analysis* | | | |
| Number of data coders | 24 | How many data coders coded the data? | 6 |
| Description of the coding tree | 25 | Did authors provide a description of the coding tree? | No |
| Derivation of themes | 26 | Were themes identified in advance or derived from the data? | No |
| Software | 27 | What software, if applicable, was used to manage the data? | n/a |
| Participant checking | 28 | Did participants provide feedback on the findings? | No |
| *Reporting* | | | |
| Quotations presented | 29 | Were participant quotations presented to illustrate the themes/findings? Was each quotation identified? e.g. participant number | 7-13 |
| Data and findings consistent | 30 | Was there consistency between the data presented and the findings? | 7-13 |
| Clarity of major themes | 31 | Were major themes clearly presented in the findings? | 7-13 |
| Clarity of minor themes | 32 | Is there a description of diverse cases or discussion of minor themes? | No |

Developed from: Tong A, Sainsbury P, Craig J. Consolidated criteria for reporting qualitative research (COREQ): a 32-item checklist for interviews and focus groups. *International Journal for Quality in Health Care*. 2007. Volume 19, Number 6: pp. 349 – 357

**Once you have completed this checklist, please save a copy and upload it as part of your submission. DO NOT include this checklist as part of the main manuscript document. It must be uploaded as a separate file.**

For peer review only

BMJ Open

# Opportunities and challenges of a novel cardiac output response to stress (CORS) test to enhance diagnosis of heart failure in primary care: a qualitative study

| | |
|---|---|
| Journal: | *BMJ Open* |
| Manuscript ID | bmjopen-2018-028122.R1 |
| Article Type: | Research |
| Date Submitted by the Author: | 12-Feb-2019 |
| Complete List of Authors: | Charman, Sarah; Newcastle University, Cardiovascular Research Centre<br>Okwose, Nduka; Newcastle University, Cardiovascular Research Centre<br>Maniatopoulos, Gregory ; Newcastle University, Institute of Health and Society<br>Graziadio, Sara; Newcastle University, NIHR In Vitro Diagnostics Co-operative<br>Metzler, Tamara; Newcastle University, Cardiovascular Research Centre<br>Banks, Helen; Newcastle University, Cardiovascular Research Centre<br>Vale, Luke; Newcastle University, Health Economics Group, Institute of Health and Society<br>MacGowan, Guy; Newcastle University, Institute of Genetic Medicine; Freeman Hospital, Cardiology<br>Seferović, Petar ; University of Belgrade School of Medicine<br>Fuat, Ahmet; Carmel Medical Practice,<br>Deaton, Christi; University of Cambridge, Cambridge Institute of Public Health, School of Clinical Medicine<br>Mant, Jonathan; University of Cambridge, General Practice and Primary Care Research Unit<br>Hobbs, Richard; University of Oxford, Dept of Primary Health Care<br>Jakovljevic, Djordje; Newcastle University, Institute for Ageing and Health |
| <b>Primary Subject Heading</b>: | Cardiovascular medicine |
| Secondary Subject Heading: | Qualitative research |
| Keywords: | Heart failure < CARDIOLOGY, Diagnosis, PRIMARY CARE, QUALITATIVE RESEARCH |
| | |

SCHOLARONE™
Manuscripts

# Opportunities and challenges of a novel cardiac output response to stress (CORS) test to enhance diagnosis of heart failure in primary care: a qualitative study

*Sarah J Charman[1, 2], *Nduka C Okwose[1, 2], Gregory Maniatopoulos[3,4], Sara Graziadio[4], Tamara Metzler[1], Helen Banks[1], Luke Vale[3], Guy A MacGowan[1,2], Petar M Seferovic[5], Ahmet Fuat[6], Christi Deaton[7], Jonathan Mant[7], F.D. Richard Hobbs[8], Djordje G Jakovljevic[1, 2 ,9]

[1] Cardiovascular Research Centre, Institutes of Cellular and Genetic Medicine, Faculty of Medical Sciences, Newcastle University, Newcastle upon Tyne, United Kingdom

[2] Cardiology Department, Freeman Hospital and Royal Victoria Infirmary, Newcastle upon Tyne Hospitals NHS Foundation Trust, Newcastle upon Tyne, United Kingdom

[3] Institute of Health and Society, Newcastle University, United Kingdom

[4] NIHR Newcastle In Vitro Diagnostics Co-operative, Newcastle upon Tyne Hospitals NHS Foundation Trust , Newcastle upon Tyne, United Kingdom

[5] Cardiology Department, Medical School, University of Belgrade, Clinical Centre Serbia and Serbian Academy of Science and Arts, Serbia

[6] Darlington Memorial Hospital, County Durham and Darlington NHS Foundation Trust & School of Medicine, Pharmacy and Health, Durham University, United Kingdom

[7] Primary Care Unit, Department of Public Health and Primary Care, University of Cambridge, Cambridge, United Kingdom

[8] Nuffield Department of Primary Health Care Sciences, University of Oxford, Oxford, United Kingdom

[9] RCUK Centre for Ageing and Vitality, Newcastle University, Newcastle Upon Tyne, United Kingdom

*Joint first authors

**Corresponding Author:** Dr Djordje Jakovljevic. Cardiovascular Research Centre, Faculty of Medical Sciences, Newcastle University, and Newcastle upon Tyne Hospitals NHS Foundation Trust, Newcastle upon Tyne, NE2 4HH, United Kingdom. Email: djordje.jakovljevic@newcastle.ac.uk. Tel: +441912088264

For peer review only



For peer review only - http://bmjopen.bmj.com/site/about/guidelines.xhtml

**Strengths and limitations of this study**

- We have recently developed a novel cardiac output response to stress (CORS) test which has potential to improve diagnostic accuracy of heart failure in primary care.

- The interviews with 14 primary and secondary health care professionals allowed exploration of the opportunities and challenges for the implementation of the CORS test.

- Study participants were recruited from one geographic region i.e. the North of England.

- No patients were interviewed.

For peer review only



For peer review only - http://bmjopen.bmj.com/site/about/guidelines.xhtml

**Abstract**

**Objective:** To explore the role of the novel Cardiac Output Response to Stress (CORS), test in the current diagnostic pathway for heart failure and the opportunities and challenges to potential implementation in primary care.

**Design:** Qualitative study using semi-structured in-depth interviews which were audio recorded and transcribed verbatim. Data from the interviews were analysed thematically using an inductive approach.

**Setting:** Newcastle upon Tyne, UK**.**

**Participants:** Fourteen healthcare professionals (six males, eight females) from primary (general practitioners, nurses, healthcare assistant, practice managers) and secondary care (consultant cardiologists).

**Results:** Four themes relating to opportunities and challenges surrounding the implementation of the new diagnostic technology were identified. These reflected that adoption of CORS test would be an advantage to primary care but the test had barriers to implementation which include: establishment of clinical utility, suitability for immobile patients and cost implication to GP practices.

**Conclusion:** The development of a simple non-invasive clinical test to accelerate the diagnosis of heart failure in primary care maybe helpful to reduce unnecessary referrals to secondary care. The CORS test has the potential to serve this purpose however, factors such as cost-effectiveness, diagnostic accuracy and seamless implementation in primary care have to be fully explored.

**Key words:** diagnosis, heart failure, primary care, qualitative research.



For peer review only - http://bmjopen.bmj.com/site/about/guidelines.xhtml

**Introduction**

Heart failure is a complex clinical syndrome resulting from impaired heart function (1, 2). It is recognised globally as a challenging public health burden and is characterised by debilitating symptoms for the patient, including breathlessness, oedema and fatigue and often co-exists with other comorbidities (3). Signs and symptoms are non-specific and therefore difficult for primary care physicians to produce an accurate diagnosis and referral (4). Increasing ageing population and improved outcomes in acute coronary syndrome have led to a growing incidence and prevalence of heart failure.

Early diagnosis of HF is crucial to improve survival, reduce hospitalisation and costs (1, 5). Primary care represents the first point of care in the clinical care pathway (figure 1) for patients presenting with symptoms of HF and General Practitioners (GPs) play a crucial role in early identification (6, 7) of patients. Evaluation of patients begin with a detailed medical history and performance of diagnostics tests. The diagnostic tests currently available to GPs include electrocardiography (ECG) (8)), and serum natriuretic peptides (NT-proBNP) tests. ECGs are insufficiently sensitive to detect heart failure and NT-proBNP test is only accurate as a test to exclude HF diagnosis however, the later test can lead to a significant number of false positives due to its low specificity (9-11). This has resulted in an unnecessary increase in referrals of patients to secondary care as >65% of suspected HF patients referred from primary to secondary care do not have diagnosis confirmed following echocardiography and specialist review (4, 8, 12, 13). This creates undue worry for the patients as there is a delay in care of patients with the average waiting time for investigation being 67 days (14). This also poses unnecessary financial and human resource burden  to secondary care providers as time and resources could have been used more effectively for patients who need the attention of secondary care specialists (1, 2, 15).

For peer review only

We have recently developed and confirmed acceptable reproducibility of a novel, easy-to-use, non-invasive cardiac output response to stress (CORS) test (Figure 2) for evaluation of cardiac function in primary care (16). The CORS test has the potential to be utilised by healthcare professionals to improve diagnostic accuracy for suspected heart failure and provide an objective measure of cardiac function as a 'rule in' test. Prior to conducting a large trial on further effectiveness of the CORS test, it was important to understand how primary and secondary healthcare professionals perceive this new diagnostic test. Information from health care providers would help us understand if there was a relevance of the CORS test and also help us make better judgement on the design of a larger trial if we thought this was plausible. The objectives of this study were firstly, to explore the role of the CORS test in the current clinical care pathway for suspected heart failure, and secondly to identify the potential opportunities and challenges to potential implementation in care of patients.

## Methods

### Study design, setting and participants

Semi-structured in-depth interviews (14 in total; Table 1) were conducted between December 2017 and April 2018 in the North of England. Participants were purposively selected according to their role and involvement in the management of patients with suspected or diagnosed heart failure in both primary and secondary care. Potential interviewees were sent an email invitation which briefly outlined the aims of the study. Those agreeing to participate were invited to recommend additional candidates for interview. Data were collected at the workplace of study participants. The study protocol (number 16/NE/0287) was approved by the National Health Service, Health Research Authority (North East – Tyne & Wear South Research Ethics Committee). All interviews were digitally recorded, anonymized and transcribed in full. One Research Associate (SJC) conducted the fieldwork.



For peer review only - http://bmjopen.bmj.com/site/about/guidelines.xhtml

Interviews were typically around 60 min in length and conducted on an individual, face-to-face basis. The final sample is described in Table 1. Firstly, participants were asked questions on the current clinical care pathway for heart failure. Then, a video demonstration of a patient completing the CORS test was shown with an explanation of each phase of the test provided by the interviewer (duration: 02:41 minutes). Finally, questions were asked about the CORS test and potential barriers and facilitators to its implementation (see appendix).

**The CORS test**

The CORS test (Figure 2) has been previously described (16) and consists of four phases: 1) rest, where the patient lies in the supine position for 3 minutes; 2) challenge, where the patient remains in the standing position for 3 minutes; 3 and 4) stress exercise, where a patient completes a step test with continuous haemodynamic measurements (i.e. cardiac output and cardiac index) using non-invasive, electrical signal processing technology.

**Patient and public involvement**

Patients/public were not involved in this study.

**Data analysis**

Given the exploratory nature of the present study, an inductive approach to data analysis was adopted. Thematic analysis was used because it is a flexible method that allowed themes to emerge freely from the data obtained (17)**.** All interviews were transcribed verbatim. Transcripts were grouped by healthcare professional role (GP, practice nurse, healthcare assistant, practice manager, consultant cardiologist) and subdivided in to primary and secondary care organisational role. The following analysis procedure was undertaken: (i) Two researchers independently read and re-read interview transcripts (SJC and NCO) in order to become adequately familiarised with the data; (ii) Both researchers independently applied codes to segments of data in all interview transcripts to develop initial themes; (iii) Due to the large volume of data generated from the interviews, several initial codes were generated and



For peer review only - http://bmjopen.bmj.com/site/about/guidelines.xhtml

For peer review only

were later cut down to a final set agreed by both researchers. The final codes were collated and this formed the basis from which themes were conceptualised. Supporting direct quotes from participants were subsequently selected for each theme. These were later discussed and approved by the research team.

**Results**

Analysis of the data generated four themes relating to opportunities and challenges surrounding the implementation of the new diagnostic technology: 1) Role of CORS test in the current pathway for HF diagnosis 2) Evidence required to improve diagnostic accuracy the of the CORS test 3) Patient exclusion and suitability/patient population for the CORS test 4) Financial Implications of the CORS test within primary care.

Theme 1: Role of CORS test in the current pathway for HF diagnosis

All GPs and consultants were very positive about the potential role of the CORS test in primary care to aid diagnosis of HF and provided reasons regarding the large numbers of undiagnosed cases of the condition.

*"Personally, I think this would be a great idea, because I think it would be, if it's more accurate, it would actually certainly reduce wastage in terms of referrals. And it would allow us to make the diagnosis much earlier and manage the patient based on that, really…. Well, you're talking to someone who believes that 70 per cent of HF is undiagnosed. And I don't know what the figure is, but most HF, I believe, is currently diagnosed in the A&E (emergency admission unit)….So, you know, you're talking to someone who thinks that anything we can do which actually makes it easier to diagnose HF in primary care is a good thing".* (GP, Practice 2).

Participants suggested that HF was difficult to diagnose with the patient often presenting with more than one complication or comorbidity, usually when disease exacerbates.



For peer review only - http://bmjopen.bmj.com/site/about/guidelines.xhtml

*“If somebody came in who had had a previous MI and said, "My ankles have swelled up. I can't lie flat in bed at night," and they had nice crackles at the bottom of their lungs, you would probably be right there and then saying, "I think this might be related to your heart," and so on. But sometimes it's much less obvious. It's just somebody presenting with breathlessness and it could be anything from heart failure to lung cancer, COPD. I mean I'm thinking of a particular patient who did end up having, in the end, quite severe HF but it wasn't obvious at all”.* (GP, Practice 3).

It was also acknowledged by secondary care participants that in primary care HF is difficult to diagnose based on the limited diagnostics available in primary care including natriuretic peptides, which can be elevated due to increasing age or other comorbidities.

*“I think you have to see quite a high proportion of people who do not have HF to avoid missing it. I also think it's quite a hard diagnosis in primary care because the blood tests are a very blunt tool. They're great for rule out but they do not rule in. Because the levels go up with age, they go up with multiple comorbidities. Many patients who are thought to have HF, actually will have a raised level, even if they turn out not to have HF”.* (Consultant Cardiologist 2, Hospital site).

One of the Consultant Cardiologists was particularly sympathetic with these concerns and acknowledged that the point of referral was different between practices and noted staff “*experience as well as knowledge and skill and confidence”* play an important role in this process.

Having a simple test like the CORS test could make identification of patients for referrals more effective, thus reducing the burden on secondary care providers. Consultants acknowledged that they often receive many inaccurate referrals which led to wasted consultant time.



For peer review only - http://bmjopen.bmj.com/site/about/guidelines.xhtml

*"That's the issue. We're seeing too many patients who we don't really need to see. If they showed me an echo and ECG [report], I would say, "I don't need to see them." But the way it's set up, it's very inefficient".* (Consultant Cardiologist 1, Hospital site).

Although, the potential value of the CORS test in primary care was clear, a variety of responses were given regarding the designated professional to deliver the CORS test i.e. GPs and nurses suggested either a practice nurse or healthcare assistant. The healthcare assistant agreed that they could deliver this test given their experience of performing ECGs but would need practice and training. Practice managers suggested that nurses could potentially be the best person to deliver it.

For Consultant Cardiologists, a qualified nurse would be the best person to administer the test in primary care but others suggested local GPs practice should guide the selection of the most appropriate person for the delivery of the CORS test.

*"you need somebody who can say, "Keep going," but you also need someone to say, "No, stop. You look absolutely awful, stop.""* (Consultant Cardiologist 2, Hospital site).

Theme 2: Evidence required to improve diagnostic accuracy the of the CORS test

Participants highlighted the role of scientific evidence in implementing a pathway for the new diagnostic technology.

*"Yes. I would want to know what's the evidence for it, how clear cut is the interpretation and actually, if this is just going to be another pre-test that is done before the people go, then what exactly is the role of the clinic".* (GP, Practice 3).

Although improvements in both referral accuracy and patient care were considered to be important for secondary care clinicians, it was difficult to determine where the CORS test would fit in the current clinical care pathway without knowing its efficacy. Secondary care



For peer review only - http://bmjopen.bmj.com/site/about/guidelines.xhtml

clinicians reported that the implementation of the new diagnostic would be less useful in secondary care with the patient already receiving an echocardiogram.

*"If it's proven to be effective in terms of being able to distinguish between possible HF or refuted diagnosis of HF, then I think it's useful. In terms of where it would fit into a pathway? Then it would appear to be that it would be at the start of the pathway before referring to secondary care."* (Consultant Cardiologist 3, Hospital site).

Successful implementation of the CORS test would be dependent upon confirmation of clinical and cost effectiveness and convincing both GP partners and nurses regarding the clinical need for it and the value of completing the test and providing good patient care.

*"You need more evidence, is the key bit. So it needs the evidence behind it. I think you might need assessment through NICE to see what they think it is as an additional test".* (Consultant Cardiologist 2, Hospital site).

Based on the comments from themes 1 and 2, it was obvious that the CORS test was vital in the primary care setting. Figure 1 shows a flow chart that describes the current clinical care pathway for the diagnosis of heart failure as adapted from the nice guidelines. The potential place of the CORS test have been inserted into the pathway to provide better context of the discussion.

Theme 3: Patient exclusion and suitability/patient population for the CORS test

A number of potential patient characteristics that could lead to patient exclusion were identified e.g. mobility, possible difficulties lying down, need of a balance aid, suggesting that not every patient would be suitable. Exercise was identified as potentially challenging. The number of patients who could potentially benefit from this technology could be increased if the duration of Phase 3 and 4 could be reduced.

*"We have some patients with established HF for example, you don't even recommend they weigh themselves every day because of the safety of stepping on to the scales. So I think there*



For peer review only - http://bmjopen.bmj.com/site/about/guidelines.xhtml

*needs that recognition that some patients may not be suitable for it."* (Consultant Cardiologist 2, Hospital site).

Other participants suggested possibility of using a set of parallel bars. GPs estimated a 50-80% rate of uptake of the CORS test while the practice nurses ware far more optimistic with an estimated 80% uptake.

Theme 4: Financial Implications of the CORS test within primary care

A major concern raised about the implementation of the CORS test was on the logistics and overall cost implication of integrating the test into routine practice. Interviewees were sceptical about the implementation of the CORS test and suggested that they would need greater convincing about the time needed to administer the test, available space, cost of equipment and the required training expected to deliver the test efficiently.

An additional appointment would be required to implement the CORS in primary care. A healthcare assistant suggested an integration of the CORS test during the current ECG and biomarker testing appointment. The practice managers acknowledged that an additional appointment will incur an additional cost to the practice but also the availability of appointments would be a potential issue.

*"Because, it wouldn't replace existing appointments [but require] additional appointments. That in itself adds a cost to the practice, that we would have to look at….That is the overall driver. You know, that assessment, obviously, has to be made at a partner and business level. The second thing, that always drives us, is cost. The third thing is impact on availability of appointments. Of course, it's availability of appointments is about the only thing as a practice we are actually measured on".* (Practice Manager, Practice 1).

Practice managers identified the space for running the tests and storage of equipment as a potential issue. Differences in the building capacity were acknowledged between practices by the practice managers. A practice nurse suggested that the receptionists would need to be aware



For peer review only - http://bmjopen.bmj.com/site/about/guidelines.xhtml

what room the equipment was in and follow similar booking process as for the ECG. On the contrary, it was suggested that a potential setting for delivery of the CORS could be at the community setting such as community diagnostic centres or outreach diagnostic facility.

*"There's increasingly, a feeling that not all practices can deliver all services at all times. However, it is more reasonable to work to the premise that all the patients of all the practices should have access to all the tests they need. But maybe just not in their own building".* (GP, Practice 2).

The cost of the equipment was also acknowledged as a potential barrier to the implementation. One approach to commissioning a new pathway is to incentivise primary care through negotiation between the primary care contractors and their Clinical Commissioning Groups.

*"I wouldn't expect them to supply it. I would expect them to fund the equipment initially. They would normally pass on subsequent maintenance costs to us. I would also expect them to commission this as an enhanced service. So, that we can recoup the manpower and equipment costs of doing it".* (Practice Manager, Practice 1).

Training was reported as a necessity by all healthcare professionals in primary care. It was suggested that not only the professional delivering the test would need to be trained, but also the professional interpreting the results. For example, a healthcare assistant could deliver the test but a GP would interpret it, in much the same process as currently occurs for ECGs.

*"From a management perspective, my real concern is also how complicated the technology is. How easy it is for a practice nurse, who may not be using it day in day out to operate it?..... You've done this test…How do you then get it to the doctor?".* (Practice Manager, Practice 1).

## Discussion



For peer review only - http://bmjopen.bmj.com/site/about/guidelines.xhtml

There has been an increase in the development and use of diagnostic tests in the last decade. This number will rise considerably taking into account the ageing population of developed countries and the number of people with chronic conditions (18). There is a clinical need to improve point of care diagnostic tests available in primary care, in particular quick and high specificity tests to diagnose HF. This would improve treatment and reduce inaccurate referrals to secondary care thus improving overall efficiency and quality of care (18). The present study captured primary and secondary care healthcare professional's perceptions for the implementation of a new diagnostic test. Our findings have identified the clinical role and potential value of the CORS test and challenges that would need to be addressed during the next phase of development of the test.

Our findings suggest that research evidence on the clinical value of the CORS test is vital before implementation. The CORS test is the first of its kind to attempt to diagnose HF in primary care and there are no studies to compare with however, we are currently assessing the sensitivity and specificity of the CORS test which will provide more information for the implementation process.

In addition, not every patient would be suitable for to CORS test due to limitation of movement. We plan to make the CORS test accessible for the majority of patients entering the clinical care pathway by shortening phase 3 and 4 and considering alternative positions (sitting or passive leg raise) using supporting aids during the exercise phase.

Similarly, implementation of the CORS test requires adequate funding and support from clinical champions and commissioners. Although there is a general consensus that point of care tests like the CORS test, increases 'turn-around- time' for results (19, 20), and prevents additional visits to secondary care (21), the financial implications of the CORS test within primary care, such as cost of tests, space, training and time need to be accounted for. These challenges which has been reported previously (22, 23) may be addressed by positioning the



For peer review only - http://bmjopen.bmj.com/site/about/guidelines.xhtml

CORS test within a community setting (i.e. community diagnostic centres or outreach diagnostic facility). This would potentially reduce the burden on the practice to deliver the test.

**Strengths and limitations**

This is the first study to explore the opportunities and challenges for the implementation of the CORS test. The perspective of primary and secondary healthcare professionals was considered, which ensured a more reflexive analysis of data (24). This approach allowed identification of suboptimal communication between primary and secondary care in the current HF clinical care pathway, which delays diagnosis and results in inaccurate referrals and wasted consultant time. Given that participants were selected only from a region of the North of England and in small numbers meant our findings may have limited generalisation.

Data were analysed thematically using an inductive approach, which led to saturation of themes at the analysis stage (25). However, this was the first iteration of the analysis of opportunities and challenges for the CORS test to inform further refinement of the CORS test.

**Future Directions**

After collection of the necessary evidence, an application to the NICE's Diagnostic Assessment Programme will be attempted to increase the possibilities of widespread implementation and adoption in primary care.

**Contributors:** *Study concept and design:* DGJ, SJC, NCO, GM, SG; *Acquisition of data:* SJC, NCO; *Data analysis and interpretation of data:* SJC, NCO, GM, SG; *Drafting of the manuscript:* SJC, GM, SG, NCO, DGJ; *Critical revision of the manuscript for important intellectual content:* TM, HB, LV, GAM, PMS, AF, CD, JM, FDRH. *Administrative, technical, or material support:* SJC, NCO; *Study Supervision:* DGJ. All authors critically reviewed the manuscript and approved the final version. DGJ acts as the guarantor and takes responsibility for the content of the manuscript, including the data and analysis.

**Funding**



For peer review only - http://bmjopen.bmj.com/site/about/guidelines.xhtml

This study was funded by the UK Medical Research Council Confidence in Concept Scheme grant to D.G.J. (grant no. BH161161). S.J.C. and N.O. are supported by the European Horizon 2020 research and innovation programme under grant agreement No 777204. S.G is supported by the NIHR MEDTEch In Vitro Diagnostics. D.G.J. is supported by the UK Research Councils' Newcastle Centre for Ageing and Vitality (grant no. L016354).The views expressed are those of the authors and not necessarily those of the Medical Research Council. The funders had no role in study design or in data collection, analysis, or interpretation.

**Ethical approval**

The study protocol (number 16/NE/0287) was approved by the National Health Service, Health Research Authority (North East – Tyne & Wear South Research Ethics Committee). All procedures performed in the study were in accordance with the Declaration of Helsinki.

**Competing interests**

The authors have no relationships or activities that could appear to have influenced the submitted work.

**Acknowledgements** We would like to thank all healthcare professionals who gave their time to being interviewed and participating in this study.

**Data sharing:** the relevant anonymised patient level data are available on reasonable request from the authors.

**Transparency:** The lead author (DGJ) affirms that the manuscript is an honest, accurate, and transparent account of the study being reported; that no important aspects of the study have been omitted; and that any discrepancies from the study as planned have been explained. The corresponding author had full access to all the study data and final responsibility for publication submission.

**Licence:** The Corresponding Author has the right to grant on behalf of all authors and does grant on behalf of all authors, a worldwide licence to the Publishers and its licensees in

perpetuity, in all forms, formats and media (whether known now or created in the future), to i) publish, reproduce, distribute, display and store the Contribution, ii) translate the Contribution into other languages, create adaptations, reprints, include within collections and create summaries, extracts and/or, abstracts of the Contribution and convert or allow conversion into any format including without limitation audio, iii) create any other derivative work(s) based in whole or part on the on the Contribution, iv) to exploit all subsidiary rights to exploit all subsidiary rights that currently exist or as may exist in the future in the Contribution, v) the inclusion of electronic links from the Contribution to third party material where-ever it may be located; and, vi) licence any third party to do any or all of the above.



For peer review only - http://bmjopen.bmj.com/site/about/guidelines.xhtml

## References


1. Ponikowski P, Voors AA, Anker SD, et al. 2016 ESC Guidelines for the diagnosis and treatment of acute and chronic heart failure: The Task Force for the diagnosis and treatment of acute and chronic heart failure of the European Society of Cardiology (ESC). Developed with the special contribution of the Heart Failure Association (HFA) of the ESC. Eur J Heart Fail. 2016;18(8):891-975.

2. National Institute for Health and Care Excellence. Chronic heart failure in adults: Diagnosis and management. In: NG106, editor. London: Royal College of Physicians; 2018.

3. National Institute for Health and Clinical Excellence. Chronic Heart Failure in Adults: Management. In: [CG108] Cg, editor. London: National Clinical Guideline Centre; 2010.

4. Fuat A, Hungin AP, Murphy JJ. Barriers to accurate diagnosis and effective management of heart failure in primary care: qualitative study. BMJ. 2003;326(7382):196.

5. Hobbs FD, Kenkre JE, Roalfe AK, et al. Impact of heart failure and left ventricular systolic dysfunction on quality of life: a cross-sectional study comparing common chronic cardiac and medical disorders and a representative adult population. Eur Heart J. 2002;23(23):1867-76.

6. Taylor CJ, Rutten FH, Brouwer JR, Hobbs FR. Practical guidance on heart failure diagnosis and management in primary care: recent EPCCS recommendations. Br J Gen Pract. 2017;67(660):326-7.

7. Roalfe AK, Mant J, Doust JA, et al. Development and initial validation of a simple clinical decision tool to predict the presence of heart failure in primary care: the MICE (Male, Infarction, Crepitations, Edema) rule. Eur J Heart Fail. 2012;14(9):1000-8.

8. Khunti K, Squire I, Abrams KR, Sutton AJ. Accuracy of a 12-lead electrocardiogram in screening patients with suspected heart failure for open access echocardiography: a systematic review and meta-analysis. Eur J Heart Fail. 2004;6(5):571-6.




For peer review only - http://bmjopen.bmj.com/site/about/guidelines.xhtml

9. Taylor CJ, Roalfe AK, Iles R, et al. Primary care REFerral for EchocaRdiogram (REFER) in heart failure: a diagnostic accuracy study. Br J Gen Pract. 2017;67(655):e94-e102.

10. Roberts E, Ludman AJ, Dworzynski K, et al. The diagnostic accuracy of the natriuretic peptides in heart failure: systematic review and diagnostic meta-analysis in the acute care setting. BMJ. 2015;350:h910.

11. Hobbs FD, Doust J, Mant J, Cowie MR. Heart failure: Diagnosis of heart failure in primary care. Heart. 2010;96(21):1773-7.

12. Kelder JC, Cramer MJ, van Wijngaarden J, et al. The diagnostic value of physical examination and additional testing in primary care patients with suspected heart failure. Circulation. 2011;124(25):2865-73.

13. Fonseca C. Diagnosis of heart failure in primary care. Heart Fail Rev. 2006;11(2):95-107.

14. Fazal IA, Bhagra SK, Bailey KM, et al. Impact of using different guideline recommended serum natriuretic peptide thresholds on the diagnosis and referral rates of a diagnostic heart failure clinic. Int J Clin Pract. 2015;69(11):1349-56.

15. Benjamin EJ, Virani SS, Callaway CW, et al. Heart Disease and Stroke Statistics-2018 Update: A Report From the American Heart Association. Circulation. 2018;137(12):e67-e492.

16. Charman SJ, Okwose NC, Stefanetti RJ, et al. A novel cardiac output response to stress test developed to improve diagnosis and monitoring of heart failure in primary care. ESC Heart Fail. 2018;5(4):703-12.

17. Braun V, Clarke V. Using thematic analysis in psychology. Qual Res Psychol. 2006;3(2):77-101.

18. Kip MMA, Hummel JM, Eppink EB, et al. Understanding the adoption and use of point-of-care tests in Dutch general practices using multi-criteria decision analysis. BMC Fam Pract. 2019;20(1):8.



For peer review only - http://bmjopen.bmj.com/site/about/guidelines.xhtml

19. Larsson A, Greig-Pylypczuk R, Huisman A. The state of point-of-care testing: a European perspective. Ups J Med Sci. 2015;120(1):1-10.

20. Laurence CO, Gialamas A, Bubner T, et al. Patient satisfaction with point-of-care testing in general practice. Br J Gen Pract. 2010;60(572):e98-104.

21. Pluddemann A, Price CP, Thompson M, Wolstenholme J, Heneghan C. Primary care diagnostic technology update: point-of-care testing for glycosylated haemoglobin. Br J Gen Pract. 2011;61(583):139-40.

22. Varzgaliene L, Heerey A, Cox C, et al. Point-of-care testing in primary care: needs and attitudes of Irish GPs. BJGP open. 2018;1(4):bjgpopen17X101229. doi: 10.3399/bjgpopen17X101229

23. Johnson M, Cross L, Sandison N, Stevenson J, Monks T, Moore M. Funding and policy incentives to encourage implementation of point-of-care C-reactive protein testing for lower respiratory tract infection in NHS primary care: a mixed-methods evaluation. BMJ open. 2018;8(10):e024558. doi: 10.1136/bmjopen-2018-024558

24. Mays N, Pope C. Qualitative research in health care. Assessing quality in qualitative research. BMJ. 2000;320(7226):50-2.

25. Saunders B, Sim J, Kingstone T, et al. Saturation in qualitative research: exploring its conceptualization and operationalization. Qual Quant. 2018;52(4):1893-907.

For peer review only



For peer review only - http://bmjopen.bmj.com/site/about/guidelines.xhtml

**List of Tables**

**Table 1:** List of interviewees.

**List of Figures**

**Figure 1:** Current clinical pathway for suspected heart failure diagnosis described by healthcare professionals in this study and adopted from the guidelines from the National Institute for Health and Care Excellence (NICE) for Chronic Heart Failure (2, 3).

**Figure 2:** Cardiac output response to stress test. The cardiac output response to stress test consists of three phases: rest, challenge, and stress exercise. Each phase lasts for 3 min, and stress phase integrates additional 3 min to increase intensity and metabolic demand (from 10 to 15 steps per minute).

**Table 1.** List of interviewees

| Organisation | No of interviews | Interviewee | Sex | Years in practice | Practice list size |
|---|---|---|---|---|---|
| Practice 1 | 2 | GP<br>Practice Manager | M<br>M | 40<br>2 | 5512 |
| Practice 2 | 3 | GP<br>Practice Nurse<br>Practice Manager | M<br>F<br>F | 15<br>15<br>2 | 5140 |
| Practice 3 | 1 | GP | F | 26 | 6491 |
| Practice 4 | 2 | GP<br>Healthcare Assistant | M<br>F | 25<br>13 | 11091 |
| Community setting | 3 | Heart Failure Specialist Nurse | F | 13, 5, 17 | |
| Hospital site | 3 | Consultant Cardiologist | F, Mx2 | 33, 30, 16 | |
| Total | 14 | | | | |



For peer review only - http://bmjopen.bmj.com/site/about/guidelines.xhtml

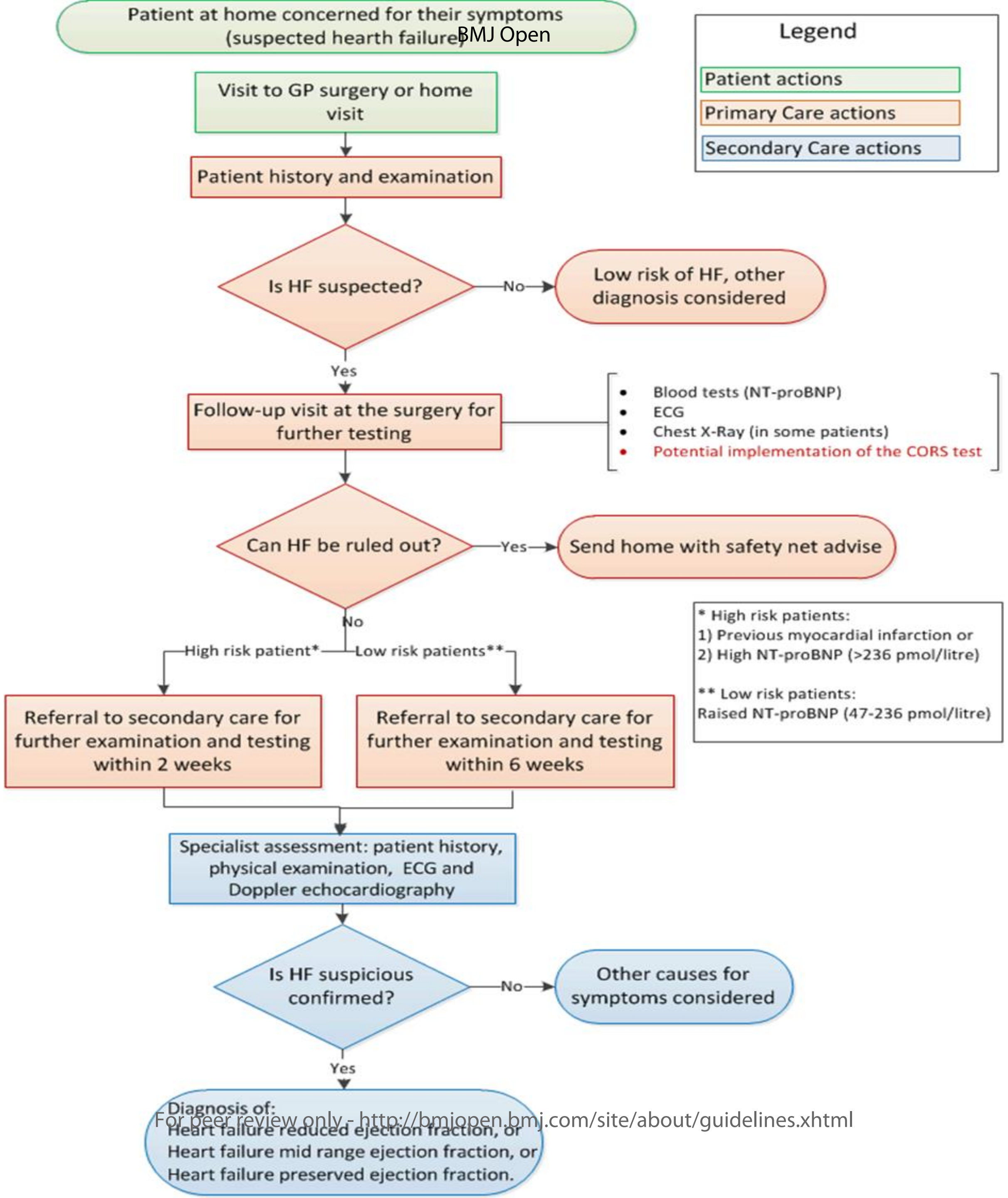
Patient at home concerned for their symptoms (suspected hearth failure)
Legend
Patient actions
Primary Care actions
Secondary Care actions
Visit to GP surgery or home visit
Patient history and examination
Is HF suspected?
No
Low risk of HF, other diagnosis considered
Yes
Follow-up visit at the surgery for further testing
• Blood tests (NT-proBNP)
• ECG
• Chest X-Ray (in some patients)
• Potential implementation of the CORS test
Can HF be ruled out?
Yes
Send home with safety net advise
No
High risk patient*
Low risk patients**
* High risk patients:
1) Previous myocardial infarction or
2) High NT-proBNP (>236 pmol/litre)
** Low risk patients:
Raised NT-proBNP (47-236 pmol/litre)
Referral to secondary care for further examination and testing within 2 weeks
Referral to secondary care for further examination and testing within 6 weeks
Specialist assessment: patient history, physical examination, ECG and Doppler echocardiography
Is HF suspicious confirmed?
No
Other causes for symptoms considered
Yes
Diagnosis of:
Heart failure reduced ejection fraction, or
Heart failure mid range ejection fraction, or
Heart failure preserved ejection fraction.

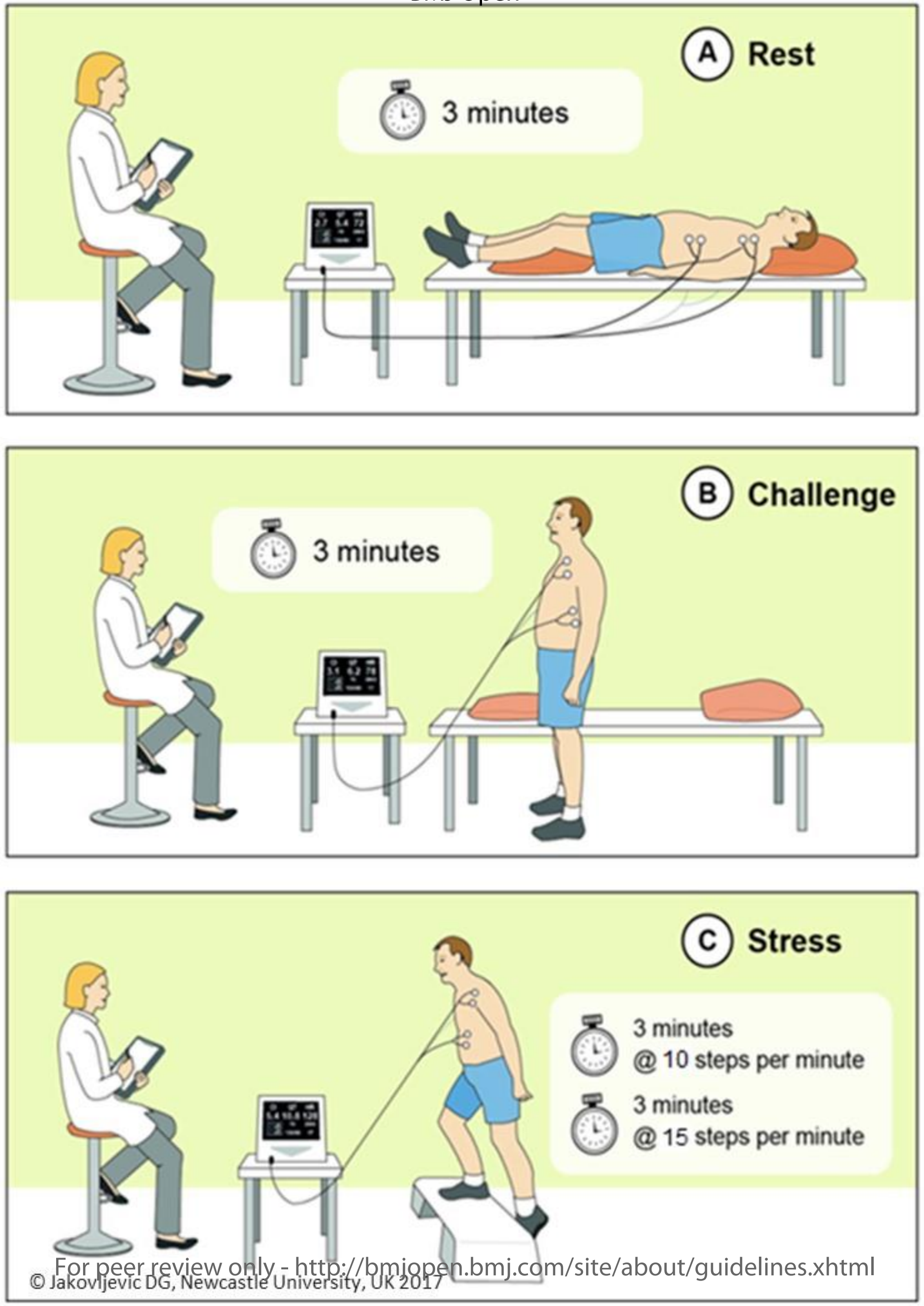


For peer review only - http://bmjopen.bmj.com/site/about/guidelines.xhtml

**Interview Schedule v1:** Consultant Cardiologists
**Project Title:** Confirmation of Acceptability of a Novel Cardiac Output Response to Stress (CORS) Test to Improve Diagnosis of Heart Failure in Primary Care

**PART 1**

**CLINICAL CARE PATHWAY**

What is the clinical care pathway for a patient with a suspected heart failure diagnosis in primary care?

When is a patient referred to secondary care?

Do the referral times from suspected heart failure diagnosis in primary care to secondary care need to be improved?

What is your role in the clinical care pathway for a patient with a suspected heart failure diagnosis?

During a heart failure diagnostic clinic, how many suspected cases of heart failure do you typically see?

How many of these cases lead to diagnosis of heart failure?

Does the current clinical care pathway lead to you seeing many patients who are unlikely to receive a heart failure diagnosis in secondary care?

How long does it take to determine a suspected heart failure case in secondary care?

Do the referral times from suspected heart failure diagnosis in primary care to confirmed diagnosis need to be improved?

For peer review only

## VIDEO OF CARDIAC OUTPUT RESPONSE TO STRESS (CORS) TEST

We are proposing a novel test which has been demonstrated to improve suspected heart failure diagnosis. This should therefore improve the number of patient referrals to secondary care.

## PART 2

## NEW CORS TEST

This novel test has been demonstrated to improve suspected heart failure diagnosis. This should therefore improve the number of patient referrals to secondary care.

What are your thoughts about this potential improvement in diagnosis being implemented in the clinical care pathway for heart failure?

What is your understanding of the new test?

Was there any information in the demonstration video that was difficult to understand?

Would a patient with a suspected heart failure diagnosis be able to complete this test?

Where in the clinical care pathway for suspected heart failure diagnosis would you expect this test to be performed?

Would you expect this test to be performed in primary or secondary care?

If this test was to become a routine part of clinical care for a patient with a suspected heart failure diagnosis, who would you expect to deliver it in primary care?

If this test was to become a routine part of clinical care for a patient with a suspected heart failure diagnosis, who would you expect to deliver it in secondary care?

Who has the greater capacity to run this test as a routine part of clinical care for a patient with a suspected heart failure diagnosis?

What problems or difficulties do you think a healthcare professional (i.e. practice nurse) running this test may encounter with a patient performing this test?

What else might be needed to deliver this test in either primary or secondary care?

What training would be needed to be able to run this test efficiently?

Do you have any further comments about running this test as part of your routine clinical care for a patient with a suspected heart failure diagnosis?

For peer review only

**Interview Schedule v1:** General Practitioners (Primary Care)

**Project Title:** Confirmation of Acceptability of a Novel Cardiac Output Response to Stress (CORS) Test to Improve Diagnosis of Heart Failure in Primary Care

**PART 1**

**CLINICAL CARE PATHWAY**

What is the clinical care pathway for a patient with a suspected heart failure diagnosis in primary care?

What is your role in the clinical care pathway for a patient with a suspected heart failure diagnosis?

What tests are performed to determine risk of heart failure?

Who would perform these tests in your practice?

How many times is a patient seen before they are referred to secondary care with suspected heart failure diagnosis?

How long is a typical consultation with a patient with a suspected heart failure diagnosis?

Who would inform a patient who was on the clinical care pathway for suspected heart failure diagnosis?

**REFERRAL**

When is a patient referred to secondary care?

What is your role in the referral process?

What are your thoughts on the referral time to secondary care?

When do you expect to receive the outcome from the referral?

What are the next steps after a patient has been diagnosed with heart failure?

What is your role in the continuing care of a patient diagnosed with heart failure?

Do the referral times from suspected heart failure diagnosis in primary care to confirmed diagnosis need to be improved?

**VIDEO OF CARDIAC OUTPUT RESPONSE TO STRESS (CORS) TEST**

We are proposing a novel test which has been demonstrated to improve suspected heart failure diagnosis. This should therefore improve the number of patient referrals to secondary care

For peer review only

## PART 2

## NEW CORS TEST

This novel test has been demonstrated to improve suspected heart failure diagnosis. This should therefore improve the number of patient referrals to secondary care.

What are your thoughts about this potential improvement in diagnosis being implemented in the clinical care pathway for heart failure?

What is your understanding of the new test?

Was there any information in the demonstration video that was difficult to understand?

Would a patient with a suspected heart failure diagnosis be able to complete this test?

Could this test be added into a routine consultation for suspected heart failure diagnosis? Or would you expect the patient need to attend a separate appointment to complete the test?

Do you anticipate any immediate barriers to implementing this test as part of your routine clinical care for a patient with a suspected heart failure diagnosis in your practice?

How easy would it be to implement this test in your practice?

If this test was to become a routine part of clinical care for a patient with a suspected heart failure diagnosis, who would you expect to deliver it?

What problems or difficulties do you think you (or the nominated colleague, i.e. practice nurse) may encounter when using this test?

What else might you need to deliver this test in practice?

What training would you (or the nominated colleague, i.e. practice nurse) need to be able to run this test?

How likely is it that this test will become part of routine clinical care for a patient with a suspected heart failure diagnosis?

Where in the clinical care pathway for suspected heart failure diagnosis would you anticipate this test being implemented?

Where do you think this test would be delivered in your practice?

Do you have the capacity to run this test as a routine part of clinical care for suspected heart failure diagnosis?

Do you have any further comments about running this test as part of your routine clinical care for a patient with a suspected heart failure diagnosis?

For peer review only

**Interview Schedule v1:** Practice Nurse (Primary Care)

**Project Title:** Confirmation of Acceptability of a Novel Cardiac Output Response to Stress (CORS) Test to Improve Diagnosis of Heart Failure in Primary Care

**PART 1**

**CLINICAL CARE PATHWAY**

What is the clinical care pathway for a patient with a suspected heart failure diagnosis in primary care?

What is your role in the clinical care pathway for a patient with a suspected heart failure diagnosis?

What tests are performed to determine risk of heart failure?

Who would perform these tests in your practice?

How long do the tests typically take?

How many times is a patient seen before they are referred to secondary care with a suspected heart failure diagnosis?

How long is a typical suspected heart failure diagnosis consultation with yourself?

Who would inform a patient who was on the clinical care pathway for suspected heart failure?

**REFERRAL**

When is a patient referred to secondary care?

Are you involved in the referral process?

What are your thoughts on the referral time to secondary care?

What are the next steps after a patient has been diagnosed with heart failure?

What is your role in the continuing care of a patient diagnosed with heart failure?

For peer review only

**VIDEO OF CARDIAC OUTPUT RESPONSE TO STRESS (CORS) TEST**

We are proposing a novel test which has been demonstrated to improve suspected heart failure diagnosis. This should therefore improve the number of patient referrals to secondary care

## PART 2

## NEW CORS TEST

This novel test has been demonstrated to improve suspected heart failure diagnosis. This should therefore improve the number of patient referrals to secondary care.

What are your thoughts about this potential improvement in diagnosis being implemented in the clinical care pathway for heart failure?

What is your understanding of the new test?

Was there any information in the demonstration video that was difficult to understand?

Would a patient with a suspected heart failure diagnosis be able to complete this test?

Could this test be added into a routine consultation for suspected heart failure diagnosis? Or would you expect the patient need to attend a separate appointment to complete the test?

Do you anticipate any immediate barriers to implementing this test as part of your routine clinical care for a patient with a suspected heart failure diagnosis?

How easy do you think it will be to implement this test in practice?

If this test was to become a routine part of clinical care for a patient suspected with a heart failure diagnosis, who would you expect to deliver it?

What problems or difficulties do you think you may encounter when using this test?

What problems or difficulties do you think you may encounter with patients performing this test?

What else might you need to deliver this test in practice?

What training would you (or a colleague) need to be able to run this test?

How confident would you be about running this test?

How likely is it that this test will become part of routine clinical care for a patient with a suspected heart failure diagnosis?

Where in the clinical care pathway for suspected heart failure diagnosis would you anticipate this test being implemented?

Where do you think this test would be delivered in your practice?

Do you have any further comments about running this test as part of your routine clinical care for a patient with a suspected heart failure diagnosis?

For peer review only

**Interview Schedule v1:** Practice Manager (Primary Care)

**Project Title:** Confirmation of Acceptability of a Novel Cardiac Output Response to Stress (CORS) Test to Improve Diagnosis of Heart Failure in Primary Care

## PART 1

## CLINICAL CARE PATHWAY

Who would perform these tests in your practice?

How many times is a patient seen before they are referred to secondary care with suspected heart failure diagnosis?

How long is a typical consultation with a patient with a suspected heart failure diagnosis?

## REFERRAL

A patient with a moderate to high NT-proBNP reading requires assessment by a specialist and echocardiogram in a heart failure diagnostic clinic in secondary care.

What is your role in the referral process?

What are your thoughts on the referral time to secondary care?

When do you expect to receive the outcome from the referral?

What are the next steps after a patient has been diagnosed with heart failure?

Do the referral times from suspected heart failure diagnosis in primary care to confirmed diagnosis need to be improved?

For peer review only

**VIDEO OF CARDIAC OUTPUT RESPONSE TO STRESS (CORS) TEST**

We are proposing a novel test which has been demonstrated to improve suspected heart failure diagnosis. This should therefore improve the number of patient referrals to secondary care

**PART 2**

**NEW CORS TEST**

This novel test has been demonstrated to improve suspected heart failure diagnosis. This should therefore improve the number of patient referrals to secondary care.

What are your thoughts about this potential improvement in diagnosis being implemented in the clinical care pathway for heart failure?

What is your understanding of the new test?

Was there any information in the demonstration video that was difficult to understand?

Would you expect commissioners to supply this equipment for each practice?

What are your thoughts on the cost of this test per patient? *Need to confirm cost per patient*

How easy would it be to implement this test in your practice?

Do you have the staff capacity to run this test as a routine part of clinical care for suspected heart failure diagnosis?

If this test was to become a routine part of clinical care for a patient with a suspected heart failure diagnosis, who would you expect to deliver it?

What problems or difficulties do you think the nominated colleague, i.e. practice nurse may encounter when using this test?

What else might you need to deliver this test in practice?

What training would the nominated colleague, i.e. practice nurse need to be able to run this test?

Where do you think this test would be delivered in your practice?

Do you have any further comments about this test becoming integrated into routine clinical care for a patient with a suspected heart failure diagnosis?

For peer review only

# COREQ (COnsolidated criteria for REporting Qualitative research) Checklist

A checklist of items that should be included in reports of qualitative research. You must report the page number in your manuscript where you consider each of the items listed in this checklist. If you have not included this information, either revise your manuscript accordingly before submitting or note N/A.

| Topic | Item No. | Guide Questions/Description | Reported on Page No. |
|---|---|---|---|
| **Domain 1: Research team and reflexivity** | | | |
| *Personal characteristics* | | | |
| Interviewer/facilitator | 1 | Which author/s conducted the interview or focus group? | 5 |
| Credentials | 2 | What were the researcher's credentials? E.g. PhD, MD | 5 |
| Occupation | 3 | What was their occupation at the time of the study? | 5 |
| Gender | 4 | Was the researcher male or female? | 5 |
| Experience and training | 5 | What experience or training did the researcher have? | 5 |
| *Relationship with participants* | | | |
| Relationship established | 6 | Was a relationship established prior to study commencement? | 5 |
| Participant knowledge of the interviewer | 7 | What did the participants know about the researcher? e.g. personal goals, reasons for doing the research | n/a |
| Interviewer characteristics | 8 | What characteristics were reported about the inter viewer/facilitator? e.g. Bias, assumptions, reasons and interests in the research topic | n/a |
| **Domain 2: Study design** | | | |
| *Theoretical framework* | | | |
| Methodological orientation and Theory | 9 | What methodological orientation was stated to underpin the study? e.g. grounded theory, discourse analysis, ethnography, phenomenology, content analysis | 5,6 |
| *Participant selection* | | | |
| Sampling | 10 | How were participants selected? e.g. purposive, convenience, consecutive, snowball | 5 |
| Method of approach | 11 | How were participants approached? e.g. face-to-face, telephone, mail, email | 5 |
| Sample size | 12 | How many participants were in the study? | 14 |
| Non-participation | 13 | How many people refused to participate or dropped out? Reasons? | n/a |
| *Setting* | | | |
| Setting of data collection | 14 | Where was the data collected? e.g. home, clinic, workplace | 5 |
| Presence of non-participants | 15 | Was anyone else present besides the participants and researchers? | no |
| Description of sample | 16 | What are the important characteristics of the sample? e.g. demographic data, date | n/a |
| *Data collection* | | | |
| Interview guide | 17 | Were questions, prompts, guides provided by the authors? Was it pilot tested? | 6 |
| Repeat interviews | 18 | Were repeat inter views carried out? If yes, how many? | no |
| Audio/visual recording | 19 | Did the research use audio or visual recording to collect the data? | 5 |
| Field notes | 20 | Were field notes made during and/or after the inter view or focus group? | No |
| Duration | 21 | What was the duration of the inter views or focus group? | 6 |
| Data saturation | 22 | Was data saturation discussed? | n/a |
| Transcripts returned | 23 | Were transcripts returned to participants for comment and/or | no |

For peer review only

| Topic | Item No. | Guide Questions/Description | Reported on Page No. |
|---|---|---|---|
| | | correction? | |
| **Domain 3: analysis and findings** | | | |
| *Data analysis* | | | |
| Number of data coders | 24 | How many data coders coded the data? | 6 |
| Description of the coding tree | 25 | Did authors provide a description of the coding tree? | No |
| Derivation of themes | 26 | Were themes identified in advance or derived from the data? | No |
| Software | 27 | What software, if applicable, was used to manage the data? | n/a |
| Participant checking | 28 | Did participants provide feedback on the findings? | No |
| *Reporting* | | | |
| Quotations presented | 29 | Were participant quotations presented to illustrate the themes/findings? Was each quotation identified? e.g. participant number | 7-13 |
| Data and findings consistent | 30 | Was there consistency between the data presented and the findings? | 7-13 |
| Clarity of major themes | 31 | Were major themes clearly presented in the findings? | 7-13 |
| Clarity of minor themes | 32 | Is there a description of diverse cases or discussion of minor themes? | No |

Developed from: Tong A, Sainsbury P, Craig J. Consolidated criteria for reporting qualitative research (COREQ): a 32-item checklist for interviews and focus groups. *International Journal for Quality in Health Care*. 2007. Volume 19, Number 6: pp. 349 – 357

**Once you have completed this checklist, please save a copy and upload it as part of your submission. DO NOT include this checklist as part of the main manuscript document. It must be uploaded as a separate file.**

For peer review only

BMJ Open

# Opportunities and challenges of a novel cardiac output response to stress (CORS) test to enhance diagnosis of heart failure in primary care: a qualitative study



| | |
|---|---|
| Journal: | *BMJ Open* |
| Manuscript ID | bmjopen-2018-028122.R2 |
| Article Type: | Research |
| Date Submitted by the Author: | 18-Mar-2019 |



| | |
|---|---|
| Complete List of Authors: | Charman, Sarah; Newcastle University, Cardiovascular Research Centre<br>Okwose, Nduka; Newcastle University, Cardiovascular Research Centre<br>Maniatopoulos, Gregory ; Newcastle University, Institute of Health and Society<br>Graziadio, Sara; Newcastle University, NIHR In Vitro Diagnostics Co-operative<br>Metzler, Tamara; Newcastle University, Cardiovascular Research Centre<br>Banks, Helen; Newcastle University, Cardiovascular Research Centre<br>Vale, Luke; Newcastle University, Health Economics Group, Institute of Health and Society<br>MacGowan, Guy; Newcastle University, Institute of Genetic Medicine; Freeman Hospital, Cardiology<br>Seferović, Petar ; University of Belgrade School of Medicine<br>Fuat, Ahmet; Carmel Medical Practice,<br>Deaton, Christi; University of Cambridge, Cambridge Institute of Public Health, School of Clinical Medicine<br>Mant, Jonathan; University of Cambridge, General Practice and Primary Care Research Unit<br>Hobbs, Richard; University of Oxford, Dept of Primary Health Care<br>Jakovljevic, Djordje; Newcastle University, Institute for Ageing and Health |



| | |
|---|---|
| <b>Primary Subject Heading</b>: | Cardiovascular medicine |
| Secondary Subject Heading: | Qualitative research |
| Keywords: | Heart failure < CARDIOLOGY, Diagnosis, PRIMARY CARE, QUALITATIVE RESEARCH |
| | |


SCHOLARONE™
Manuscripts

# Opportunities and challenges of a novel cardiac output response to stress (CORS) test to enhance diagnosis of heart failure in primary care: a qualitative study

*Sarah J Charman[1, 2], *Nduka C Okwose[1, 2], Gregory Maniatopoulos[3,4], Sara Graziadio[4], Tamara Metzler[1], Helen Banks[1], Luke Vale[3], Guy A MacGowan[1,2], Petar M Seferovic[5], Ahmet Fuat[6], Christi Deaton[7], Jonathan Mant[7], F.D. Richard Hobbs[8], Djordje G Jakovljevic[1, 2 ,9]

[1] Cardiovascular Research Centre, Institutes of Cellular and Genetic Medicine, Faculty of Medical Sciences, Newcastle University, Newcastle upon Tyne, United Kingdom

[2] Cardiology Department, Freeman Hospital and Royal Victoria Infirmary, Newcastle upon Tyne Hospitals NHS Foundation Trust, Newcastle upon Tyne, United Kingdom

[3] Institute of Health and Society, Newcastle University, United Kingdom

[4] NIHR Newcastle In Vitro Diagnostics Co-operative, Newcastle upon Tyne Hospitals NHS Foundation Trust , Newcastle upon Tyne, United Kingdom

[5] Cardiology Department, Medical School, University of Belgrade, Clinical Centre Serbia and Serbian Academy of Science and Arts, Serbia

[6] Darlington Memorial Hospital, County Durham and Darlington NHS Foundation Trust & School of Medicine, Pharmacy and Health, Durham University, United Kingdom

[7] Primary Care Unit, Department of Public Health and Primary Care, University of Cambridge, Cambridge, United Kingdom

[8] Nuffield Department of Primary Health Care Sciences, University of Oxford, Oxford, United Kingdom

[9] RCUK Centre for Ageing and Vitality, Newcastle University, Newcastle Upon Tyne, United Kingdom

*Joint first authors

**Corresponding Author:** Dr Djordje Jakovljevic. Cardiovascular Research Centre, Faculty of Medical Sciences, Newcastle University, and Newcastle upon Tyne Hospitals NHS Foundation Trust, Newcastle upon Tyne, NE2 4HH, United Kingdom. Email: djordje.jakovljevic@newcastle.ac.uk. Tel: +441912088264

For peer review only

**Strengths and limitations of this study**

- We have recently developed a novel cardiac output response to stress (CORS) test which has potential to improve diagnostic accuracy of heart failure in primary care.

- The interviews with 14 primary and secondary health care professionals allowed exploration of the opportunities and challenges for the implementation of the CORS test.

- Study participants were recruited from one geographic region i.e. the North of England.

- No patients were interviewed.

For peer review only



For peer review only - http://bmjopen.bmj.com/site/about/guidelines.xhtml

## Abstract

**Objective:** To explore the role of the novel Cardiac Output Response to Stress (CORS), test in the current diagnostic pathway for heart failure and the opportunities and challenges to potential implementation in primary care.

**Design:** Qualitative study using semi-structured in-depth interviews which were audio recorded and transcribed verbatim. Data from the interviews were analysed thematically using an inductive approach.

**Setting:** Newcastle upon Tyne, UK**.**

**Participants:** Fourteen healthcare professionals (six males, eight females) from primary (general practitioners, nurses, healthcare assistant, practice managers) and secondary care (consultant cardiologists).

**Results:** Four themes relating to opportunities and challenges surrounding the implementation of the new diagnostic technology were identified. These reflected that adoption of CORS test would be an advantage to primary care but the test had barriers to implementation which include: establishment of clinical utility, suitability for immobile patients and cost implication to GP practices.

**Conclusion:** The development of a simple non-invasive clinical test to accelerate the diagnosis of heart failure in primary care maybe helpful to reduce unnecessary referrals to secondary care. The CORS test has the potential to serve this purpose however, factors such as cost-effectiveness, diagnostic accuracy and seamless implementation in primary care have to be fully explored.


**Key words:** diagnosis, heart failure, primary care, qualitative research.



For peer review only - http://bmjopen.bmj.com/site/about/guidelines.xhtml

## Introduction

Heart failure is a complex clinical syndrome resulting from impaired heart function (1, 2). It is recognised globally as a challenging public health burden and is characterised by debilitating symptoms for the patient, including breathlessness, oedema and fatigue and often co-exists with other comorbidities (3). Signs and symptoms are non-specific and therefore difficult for primary care physicians to produce an accurate diagnosis and referral (4). Increasing ageing population and improved outcomes in acute coronary syndrome have led to a growing incidence and prevalence of heart failure.

Early diagnosis of HF is crucial to improve survival, reduce hospitalisation and costs (1, 5). Primary care represents the first point of care in the clinical care pathway (figure 1) for patients presenting with symptoms of HF and General Practitioners (GPs) play a crucial role in early identification (6, 7) of patients. Evaluation of patients begin with a detailed medical history and performance of diagnostics tests. The diagnostic tests currently available to GPs include electrocardiography (ECG) (8)), and serum natriuretic peptides (NT-proBNP) tests. ECGs are insufficiently sensitive to detect heart failure and NT-proBNP test is only accurate as a test to exclude HF diagnosis however, the later test can lead to a significant number of false positives due to its low specificity (9-11). This has resulted in an unnecessary increase in referrals of patients to secondary care as >65% of suspected HF patients referred from primary to secondary care do not have diagnosis confirmed following echocardiography and specialist review (4, 8, 12, 13). This creates undue worry for the patients as there is a delay in care of patients with the average waiting time for investigation being 67 days (14). This also poses unnecessary financial and human resource burden  to secondary care providers as time and resources could have been used more effectively for patients who need the attention of secondary care specialists (1, 2, 15).



For peer review only - http://bmjopen.bmj.com/site/about/guidelines.xhtml

We have recently developed and confirmed acceptable reproducibility of a novel, easy-to-use, non-invasive cardiac output response to stress (CORS) test (Figure 2) for evaluation of cardiac function in primary care (16). The CORS test has the potential to be utilised by healthcare professionals to improve diagnostic accuracy for suspected heart failure and provide an objective measure of cardiac function as a 'rule in' test. Prior to conducting a large trial on further effectiveness of the CORS test, it was important to understand how primary and secondary healthcare professionals perceive this new diagnostic test. Information from health care providers would help us understand if there was a relevance of the CORS test and also help us make better judgement on the design of a larger trial if we thought this was plausible. The objectives of this study were firstly, to explore the role of the CORS test in the current clinical care pathway for suspected heart failure, and secondly to identify the potential opportunities and challenges to potential implementation in care of patients.

## Methods

### Study design, setting and participants

Semi-structured in-depth interviews (14 in total; Table 1) were conducted between December 2017 and April 2018 in the North of England. Participants were purposely selected according to their role and involvement in the management of patients with suspected or diagnosed heart failure in both primary and secondary care. Potential interviewees were sent an email invitation which briefly outlined the aims of the study. Those agreeing to participate were invited to recommend additional candidates for interview. Data were collected at the workplace of study participants. The study protocol (number 16/NE/0287) was approved by the National Health Service, Health Research Authority (North East – Tyne & Wear South Research Ethics Committee). All interviews were digitally recorded, anonymized and transcribed in full.

One Research Associate (SJC) conducted the fieldwork.



For peer review only - http://bmjopen.bmj.com/site/about/guidelines.xhtml

Interviews were typically around 60 min in length and conducted on an individual, face-to-face basis. The final sample is described in Table 1. Firstly, participants were asked questions on the current clinical care pathway for heart failure. Then, a video demonstration of a patient completing the CORS test was shown with an explanation of each phase of the test provided by the interviewer (duration: 02:41 minutes). Finally, questions were asked about the CORS test and potential barriers and facilitators to its implementation. Examples of questions asked which relate to the main objectives of the study include: 1) Does the current clinical care pathway lead to you seeing many patients who are unlikely to receive a heart failure diagnosis in secondary care?; 2) Do the referral time from suspected heart failure diagnosis in primary care to confirmed diagnosis need to be improved?; 3) What are your thoughts about this potential improvement in diagnosis being implemented in the clinical care pathway for heart failure?; 4) Where in the clinical care pathway for suspected heart failure diagnosis would you expect this test to be performed?; 5) Do you anticipate any immediate barriers to implementing this test as part of your routine clinical care for a patient with a suspected heart failure diagnosis in your practice?. Prompts were used generate a deeper understanding of participants' views about the questions asked (please see appendix for full list of interview questions and prompts).

**The CORS test**

The CORS test (Figure 2) has been previously described (16) and consists of four phases: 1) rest, where the patient lies in the supine position for 3 minutes; 2) challenge, where the patient remains in the standing position for 3 minutes; 3 and 4) stress exercise, where a patient completes a step test with continuous haemodynamic measurements (i.e. cardiac output and cardiac index) using non-invasive, electrical signal processing technology.



For peer review only - http://bmjopen.bmj.com/site/about/guidelines.xhtml

**Patient and public involvement**

Patients/public representatives were not directly involved in the present qualitative study. However, their views about the design of the main study which aims to assess the clinical- and cost-effectiveness of the CORS test to improve diagnosis of heart failure in primary care, have been explored.

**Data analysis**

Given the exploratory nature of the present study, an inductive approach to data analysis was adopted. Thematic analysis was used because it is a flexible method that allowed themes to emerge freely from the data obtained (17). All interviews were transcribed verbatim. The following analysis procedure was undertaken: (i) Two researchers independently read and re-read interview transcripts (SJC and NCO) in order to become adequately familiarised with the data; (ii) Both researchers independently applied codes to segments of data in all interview transcripts to develop initial themes; (iii) Due to the large volume of data generated from the interviews, several initial codes were generated and were later cut down to a final set agreed by both researchers. The final codes were collated and this formed the basis from which themes were conceptualised. Supporting direct quotes from participants were subsequently selected for each theme. These were later discussed and approved by the research team.

**Results**

Analysis of the data generated four themes relating to opportunities and challenges surrounding the implementation of the new diagnostic technology: 1) Novel diagnostic tests are required to improve HF diagnosis; 2) Evidence required to improve diagnostic accuracy the of the CORS test; 3) Patient exclusion and suitability/patient population for the CORS test; and 4) Financial Implications of the CORS test within primary care.

Theme 1: Novel diagnostic tests are required to improve HF diagnosis



For peer review only - http://bmjopen.bmj.com/site/about/guidelines.xhtml

All GPs and consultants agreed that new diagnostic tests vital to improve HF diagnosis and were very positive about the potential role of the CORS test in primary care to aid diagnosis of HF and provided reasons regarding the large numbers of undiagnosed cases of the condition.

*"Personally, I think this would be a great idea, because I think it would be, if it's more accurate, it would actually certainly reduce wastage in terms of referrals. And it would allow us to make the diagnosis much earlier and manage the patient based on that, really…. Well, you're talking to someone who believes that 70 per cent of HF is undiagnosed. And I don't know what the figure is, but most HF, I believe, is currently diagnosed in the A&E (emergency admission unit)….So, you know, you're talking to someone who thinks that anything we can do which actually makes it easier to diagnose HF in primary care is a good thing".* (GP, Practice 2).

Participants suggested that HF was difficult to diagnose with the patient often presenting with more than one complication or comorbidity, usually when disease exacerbates.

*"If somebody came in who had had a previous MI and said, "My ankles have swelled up. I can't lie flat in bed at night," and they had nice crackles at the bottom of their lungs, you would probably be right there and then saying, "I think this might be related to your heart," and so on. But sometimes it's much less obvious. It's just somebody presenting with breathlessness and it could be anything from heart failure to lung cancer, COPD. I mean I'm thinking of a particular patient who did end up having, in the end, quite severe HF but it wasn't obvious at all".* (GP, Practice 3).

It was also acknowledged by secondary care participants that in primary care HF is difficult to diagnose based on the limited diagnostics available in primary care including natriuretic peptides, which can be elevated due to increasing age or other comorbidities.

*"I think you have to see quite a high proportion of people who do not have HF to avoid missing it. I also think it's quite a hard diagnosis in primary care because the blood tests are a very*



For peer review only - http://bmjopen.bmj.com/site/about/guidelines.xhtml

*blunt tool. They're great for rule out but they do not rule in. Because the levels go up with age, they go up with multiple comorbidities. Many patients who are thought to have HF, actually will have a raised level, even if they turn out not to have HF".* (Consultant Cardiologist 2, Hospital site).

One of the Consultant Cardiologists was particularly sympathetic with these concerns and acknowledged that the point of referral was different between practices and noted staff "*experience as well as knowledge and skill and confidence"* play an important role in this process.

Having a simple test like the CORS test could make identification of patients for referrals more effective, thus reducing the burden on secondary care providers. Consultants acknowledged that they often receive many inaccurate referrals which led to wasted consultant time.

*"That's the issue. We're seeing too many patients who we don't really need to see. If they showed me an echo and ECG [report], I would say, "I don't need to see them." But the way it's set up, it's very inefficient".* (Consultant Cardiologist 1, Hospital site).

Although, the potential value of the CORS test in primary care was clear, a variety of responses were given regarding the designated professional to deliver the CORS test i.e. GPs and nurses suggested either a practice nurse or healthcare assistant. The healthcare assistant agreed that they could deliver this test given their experience of performing ECGs but would need practice and training. Practice managers suggested that nurses could potentially be the best person to deliver it.

For Consultant Cardiologists, a qualified nurse would be the best person to administer the test in primary care but others suggested local GPs practice should guide the selection of the most appropriate person for the delivery of the CORS test.

*"you need somebody who can say, "Keep going," but you also need someone to say, "No, stop. You look absolutely awful, stop.""* (Consultant Cardiologist 2, Hospital site).



For peer review only - http://bmjopen.bmj.com/site/about/guidelines.xhtml

Theme 2: Evidence required to improve diagnostic accuracy the of the CORS test

Participants highlighted the role of scientific evidence in implementing a pathway for the new diagnostic technology.

*"Yes. I would want to know what's the evidence for it, how clear cut is the interpretation and actually, if this is just going to be another pre-test that is done before the people go, then what exactly is the role of the clinic".* (GP, Practice 3).

Although improvements in both referral accuracy and patient care were considered to be important for secondary care clinicians, it was difficult to determine where the CORS test would fit in the current clinical care pathway without knowing its efficacy. Secondary care clinicians reported that the implementation of the new diagnostic would be less useful in secondary care with the patient already receiving an echocardiogram.

*"If it's proven to be effective in terms of being able to distinguish between possible HF or refuted diagnosis of HF, then I think it's useful. In terms of where it would fit into a pathway? Then it would appear to be that it would be at the start of the pathway before referring to secondary care."* (Consultant Cardiologist 3, Hospital site).

Successful implementation of the CORS test would be dependent upon confirmation of clinical and cost effectiveness and convincing both GP partners and nurses regarding the clinical need for it and the value of completing the test and providing good patient care.

*"You need more evidence, is the key bit. So it needs the evidence behind it. I think you might need assessment through NICE to see what they think it is as an additional test".* (Consultant Cardiologist 2, Hospital site).

Based on the comments from themes 1 and 2, it was obvious that the CORS test was vital in the primary care setting. Figure 1 shows a flow chart that describes the current clinical care pathway for the diagnosis of heart failure as adapted from the nice guidelines. The potential



For peer review only - http://bmjopen.bmj.com/site/about/guidelines.xhtml

place of the CORS test have been inserted into the pathway to provide better context of the discussion.

Theme 3: Patient exclusion and suitability/patient population for the CORS test

A number of potential patient characteristics that could lead to patient exclusion were identified e.g. mobility, possible difficulties lying down, need of a balance aid, suggesting that not every patient would be suitable. Exercise was identified as potentially challenging. The number of patients who could potentially benefit from this technology could be increased if the duration of Phase 3 and 4 could be reduced.

*"We have some patients with established HF for example, you don't even recommend they weigh themselves every day because of the safety of stepping on to the scales. So I think there needs that recognition that some patients may not be suitable for it."* (Consultant Cardiologist 2, Hospital site).

Other participants suggested possibility of using a set of parallel bars. GPs estimated a 50-80% rate of uptake of the CORS test while the practice nurses ware far more optimistic with an estimated 80% uptake.

Theme 4: Financial Implications of the CORS test within primary care

A major concern raised about the implementation of the CORS test was on the logistics and overall cost implication of integrating the test into routine practice. Interviewees were sceptical about the implementation of the CORS test and suggested that they would need greater convincing about the time needed to administer the test, available space, cost of equipment and the required training expected to deliver the test efficiently.

An additional appointment would be required to implement the CORS in primary care. A healthcare assistant suggested an integration of the CORS test during the current ECG and biomarker testing appointment. The practice managers acknowledged that an additional



For peer review only - http://bmjopen.bmj.com/site/about/guidelines.xhtml

appointment will incur an additional cost to the practice but also the availability of appointments would be a potential issue.

*"Because, it wouldn't replace existing appointments [but require] additional appointments. That in itself adds a cost to the practice, that we would have to look at….That is the overall driver. You know, that assessment, obviously, has to be made at a partner and business level. The second thing, that always drives us, is cost. The third thing is impact on availability of appointments. Of course, it's availability of appointments is about the only thing as a practice we are actually measured on".* (Practice Manager, Practice 1).

Practice managers identified the space for running the tests and storage of equipment as a potential issue. Differences in the building capacity were acknowledged between practices by the practice managers. A practice nurse suggested that the receptionists would need to be aware what room the equipment was in and follow similar booking process as for the ECG. On the contrary, it was suggested that a potential setting for delivery of the CORS could be at the community setting such as community diagnostic centres or outreach diagnostic facility.

*"There's increasingly, a feeling that not all practices can deliver all services at all times. However, it is more reasonable to work to the premise that all the patients of all the practices should have access to all the tests they need. But maybe just not in their own building".* (GP, Practice 2).

The cost of the equipment was also acknowledged as a potential barrier to the implementation. One approach to commissioning a new pathway is to incentivise primary care through negotiation between the primary care contractors and their Clinical Commissioning Groups.

*"I wouldn't expect them to supply it. I would expect them to fund the equipment initially. They would normally pass on subsequent maintenance costs to us. I would also expect them to commission this as an enhanced service. So, that we can recoup the manpower and equipment costs of doing it".* (Practice Manager, Practice 1).



For peer review only - http://bmjopen.bmj.com/site/about/guidelines.xhtml

Training was reported as a necessity by all healthcare professionals in primary care. It was suggested that not only the professional delivering the test would need to be trained, but also the professional interpreting the results. For example, a healthcare assistant could deliver the test but a GP would interpret it, in much the same process as currently occurs for ECGs.

*"From a management perspective, my real concern is also how complicated the technology is. How easy it is for a practice nurse, who may not be using it day in day out to operate it?..... You've done this test...How do you then get it to the doctor?".* (Practice Manager, Practice 1).

For peer review only

**Discussion**

There has been an increase in the development and use of diagnostic tests in the last decade. This number will rise considerably taking into account the ageing population of developed countries and the number of people with chronic conditions (18). There is a clinical need to improve point of care diagnostic tests available in primary care, in particular quick and high specificity tests to diagnose HF. This would improve treatment and reduce inaccurate referrals to secondary care thus improving overall efficiency and quality of care (18). The present study captured primary and secondary care healthcare professional's perceptions for the implementation of a new diagnostic test. Our findings have identified the clinical role and potential value of the CORS test and challenges that would need to be addressed during the next phase of development of the test.

Our findings suggest that research evidence on the clinical value of the CORS test is vital before implementation. The CORS test is the first of its kind to attempt to diagnose HF in primary care and there are no studies to compare with however, we are currently assessing the sensitivity and specificity of the CORS test which will provide more information for the implementation process.



For peer review only - http://bmjopen.bmj.com/site/about/guidelines.xhtml

In addition, not every patient would be suitable for to CORS test due to limitation of movement. We plan to make the CORS test accessible for the majority of patients entering the clinical care pathway by shortening phase 3 and 4 and considering alternative positions (sitting or passive leg raise) using supporting aids during the exercise phase.

Similarly, implementation of the CORS test requires adequate funding and support from clinical champions and commissioners. Although there is a general consensus that point of care tests like the CORS test, increases 'turn-around- time' for results (19, 20), and prevents additional visits to secondary care (21), the financial implications of the CORS test within primary care, such as cost of tests, space, training and time need to be accounted for. These challenges which has been reported previously (22, 23) may be addressed by positioning the CORS test within a community setting (i.e. community diagnostic centres or outreach diagnostic facility). This would potentially reduce the burden on the practice to deliver the test.

**Strengths and limitations**

This is the first study to explore the opportunities and challenges for the implementation of the CORS test. The perspective of primary and secondary healthcare professionals was considered, which ensured a more reflexive analysis of data (24). This approach allowed identification of suboptimal communication between primary and secondary care in the current HF clinical care pathway, which delays diagnosis and results in inaccurate referrals and wasted consultant time. Given that participants were selected only from a region of the North of England and in small numbers meant our findings may have limited generalisation.

Data were analysed thematically using an inductive approach, which led to saturation of themes at the analysis stage (25). However, this was the first iteration of the analysis of opportunities and challenges for the CORS test to inform further refinement of the CORS test.

**Future Directions**



For peer review only - http://bmjopen.bmj.com/site/about/guidelines.xhtml

After collection of the necessary evidence, an application to the NICE's Diagnostic Assessment Programme will be attempted to increase the possibilities of widespread implementation and adoption in primary care.

**Contributors:** *Study concept and design:* DGJ, SJC, NCO, GM, SG; *Acquisition of data:* SJC, NCO; *Data analysis and interpretation of data:* SJC, NCO, GM, SG; *Drafting of the manuscript:* SJC, GM, SG, NCO, DGJ; *Critical revision of the manuscript for important intellectual content:* TM, HB, LV, GAM, PMS, AF, CD, JM, FDRH. *Administrative, technical, or material support:* SJC, NCO; *Study Supervision:* DGJ. All authors critically reviewed the manuscript and approved the final version. DGJ acts as the guarantor and takes responsibility for the content of the manuscript, including the data and analysis.

**Funding**

This study was funded by the UK Medical Research Council Confidence in Concept Scheme grant to D.G.J. (grant no. BH161161). S.J.C. and N.O. are supported by the European Horizon 2020 research and innovation programme under grant agreement No 777204. S.G is supported by the NIHR MEDTEch In Vitro Diagnostics. D.G.J. is supported by the UK Research Councils' Newcastle Centre for Ageing and Vitality (grant no. L016354).The views expressed are those of the authors and not necessarily those of the Medical Research Council. The funders had no role in study design or in data collection, analysis, or interpretation.

**Ethical approval**

The study protocol (number 16/NE/0287) was approved by the National Health Service, Health Research Authority (North East – Tyne & Wear South Research Ethics Committee). All procedures performed in the study were in accordance with the Declaration of Helsinki.

**Competing interests**

The authors have no relationships or activities that could appear to have influenced the submitted work.



For peer review only - http://bmjopen.bmj.com/site/about/guidelines.xhtml

**Acknowledgements** We would like to thank all healthcare professionals who gave their time to being interviewed and participating in this study.

**Data sharing:** the relevant anonymised patient level data are available on reasonable request from the authors.

**Transparency:** The lead author (DGJ) affirms that the manuscript is an honest, accurate, and transparent account of the study being reported; that no important aspects of the study have been omitted; and that any discrepancies from the study as planned have been explained. The corresponding author had full access to all the study data and final responsibility for publication submission.

**Licence:** The Corresponding Author has the right to grant on behalf of all authors and does grant on behalf of all authors, a worldwide licence to the Publishers and its licensees in perpetuity, in all forms, formats and media (whether known now or created in the future), to i) publish, reproduce, distribute, display and store the Contribution, ii) translate the Contribution into other languages, create adaptations, reprints, include within collections and create summaries, extracts and/or, abstracts of the Contribution and convert or allow conversion into any format including without limitation audio, iii) create any other derivative work(s) based in whole or part on the on the Contribution, iv) to exploit all subsidiary rights to exploit all subsidiary rights that currently exist or as may exist in the future in the Contribution, v) the inclusion of electronic links from the Contribution to third party material where-ever it may be located; and, vi) licence any third party to do any or all of the above.

## References

1. Ponikowski P, Voors AA, Anker SD, et al. 2016 ESC Guidelines for the diagnosis and treatment of acute and chronic heart failure: The Task Force for the diagnosis and treatment of acute and chronic heart failure of the European Society of Cardiology (ESC). Developed with the special contribution of the Heart Failure Association (HFA) of the ESC. Eur J Heart Fail. 2016;18(8):891-975.

2. National Institute for Health and Care Excellence. Chronic heart failure in adults: Diagnosis and management. In: NG106, editor. London: Royal College of Physicians; 2018.

3. National Institute for Health and Clinical Excellence. Chronic Heart Failure in Adults: Management. In: [CG108] Cg, editor. London: National Clinical Guideline Centre; 2010.

4. Fuat A, Hungin AP, Murphy JJ. Barriers to accurate diagnosis and effective management of heart failure in primary care: qualitative study. BMJ. 2003;326(7382):196.

5. Hobbs FD, Kenkre JE, Roalfe AK, et al. Impact of heart failure and left ventricular systolic dysfunction on quality of life: a cross-sectional study comparing common chronic cardiac and medical disorders and a representative adult population. Eur Heart J. 2002;23(23):1867-76.

6. Taylor CJ, Rutten FH, Brouwer JR, Hobbs FR. Practical guidance on heart failure diagnosis and management in primary care: recent EPCCS recommendations. Br J Gen Pract. 2017;67(660):326-7.

For peer review only

7. Roalfe AK, Mant J, Doust JA, et al. Development and initial validation of a simple clinical decision tool to predict the presence of heart failure in primary care: the MICE (Male, Infarction, Crepitations, Edema) rule. Eur J Heart Fail. 2012;14(9):1000-8.

8. Khunti K, Squire I, Abrams KR, Sutton AJ. Accuracy of a 12-lead electrocardiogram in screening patients with suspected heart failure for open access echocardiography: a systematic review and meta-analysis. Eur J Heart Fail. 2004;6(5):571-6.

9. Taylor CJ, Roalfe AK, Iles R, et al. Primary care REFerral for EchocaRdiogram (REFER) in heart failure: a diagnostic accuracy study. Br J Gen Pract. 2017;67(655):e94-e102.

10. Roberts E, Ludman AJ, Dworzynski K, et al. The diagnostic accuracy of the natriuretic peptides in heart failure: systematic review and diagnostic meta-analysis in the acute care setting. BMJ. 2015;350:h910.

11. Hobbs FD, Doust J, Mant J, Cowie MR. Heart failure: Diagnosis of heart failure in primary care. Heart. 2010;96(21):1773-7.

12. Kelder JC, Cramer MJ, van Wijngaarden J, et al. The diagnostic value of physical examination and additional testing in primary care patients with suspected heart failure. Circulation. 2011;124(25):2865-73.

13. Fonseca C. Diagnosis of heart failure in primary care. Heart Fail Rev. 2006;11(2):95-107.

14. Fazal IA, Bhagra SK, Bailey KM, et al. Impact of using different guideline recommended serum natriuretic peptide thresholds on the diagnosis and referral rates of a diagnostic heart failure clinic. Int J Clin Pract. 2015;69(11):1349-56.

15. Benjamin EJ, Virani SS, Callaway CW, et al. Heart Disease and Stroke Statistics-2018 Update: A Report From the American Heart Association. Circulation. 2018;137(12):e67-e492.

For peer review only



For peer review only - http://bmjopen.bmj.com/site/about/guidelines.xhtml

16. Charman SJ, Okwose NC, Stefanetti RJ, et al. A novel cardiac output response to stress test developed to improve diagnosis and monitoring of heart failure in primary care. ESC Heart Fail. 2018;5(4):703-12.

17. Braun V, Clarke V. Using thematic analysis in psychology. Qual Res Psychol. 2006;3(2):77-101.

18. Kip MMA, Hummel JM, Eppink EB, et al. Understanding the adoption and use of point-of-care tests in Dutch general practices using multi-criteria decision analysis. BMC Fam Pract. 2019;20(1):8.

19. Larsson A, Greig-Pylypczuk R, Huisman A. The state of point-of-care testing: a European perspective. Ups J Med Sci. 2015;120(1):1-10.

20. Laurence CO, Gialamas A, Bubner T, et al. Patient satisfaction with point-of-care testing in general practice. Br J Gen Pract. 2010;60(572):e98-104.

21. Pluddemann A, Price CP, Thompson M, Wolstenholme J, Heneghan C. Primary care diagnostic technology update: point-of-care testing for glycosylated haemoglobin. Br J Gen Pract. 2011;61(583):139-40.

22. Varzgaliene L, Heerey A, Cox C, et al. Point-of-care testing in primary care: needs and attitudes of Irish GPs. BJGP open. 2018;1(4):bjgpopen17X101229. doi: 10.3399/bjgpopen17X101229

23. Johnson M, Cross L, Sandison N, Stevenson J, Monks T, Moore M. Funding and policy incentives to encourage implementation of point-of-care C-reactive protein testing for lower respiratory tract infection in NHS primary care: a mixed-methods evaluation. BMJ open. 2018;8(10):e024558. doi: 10.1136/bmjopen-2018-024558

24. Mays N, Pope C. Qualitative research in health care. Assessing quality in qualitative research. BMJ. 2000;320(7226):50-2.



For peer review only - http://bmjopen.bmj.com/site/about/guidelines.xhtml

25. Saunders B, Sim J, Kingstone T, et al. Saturation in qualitative research: exploring its conceptualization and operationalization. Qual Quant. 2018;52(4):1893-907.

For peer review only

**List of Tables**

**Table 1:** List of interviewees.

**List of Figures**

**Figure 1:** Current clinical pathway for suspected heart failure diagnosis described by healthcare professionals in this study and adopted from the guidelines from the National Institute for Health and Care Excellence (NICE) for Chronic Heart Failure (2, 3).

**Figure 2:** Cardiac output response to stress test. The cardiac output response to stress test consists of three phases: rest, challenge, and stress exercise. Each phase lasts for 3 min, and stress phase integrates additional 3 min to increase intensity and metabolic demand (from 10 to 15 steps per minute).

**Table 1.** List of interviewees

| Organisation | No of interviews | Interviewee | Sex | Years in practice | Practice list size |
|---|---|---|---|---|---|
| Practice 1 | 2 | GP<br>Practice Manager | M<br>M | 40<br>2 | 5512 |
| Practice 2 | 3 | GP<br>Practice Nurse<br>Practice Manager | M<br>F<br>F | 15<br>15<br>2 | 5140 |
| Practice 3 | 1 | GP | F | 26 | 6491 |
| Practice 4 | 2 | GP<br>Healthcare Assistant | M<br>F | 25<br>13 | 11091 |
| Community setting | 3 | Heart Failure Specialist Nurse | F | 13, 5, 17 | |
| Hospital site | 3 | Consultant Cardiologist | F, Mx2 | 33, 30, 16 | |
| Total | 14 | | | | |



For peer review only - http://bmjopen.bmj.com/site/about/guidelines.xhtml

For peer review only

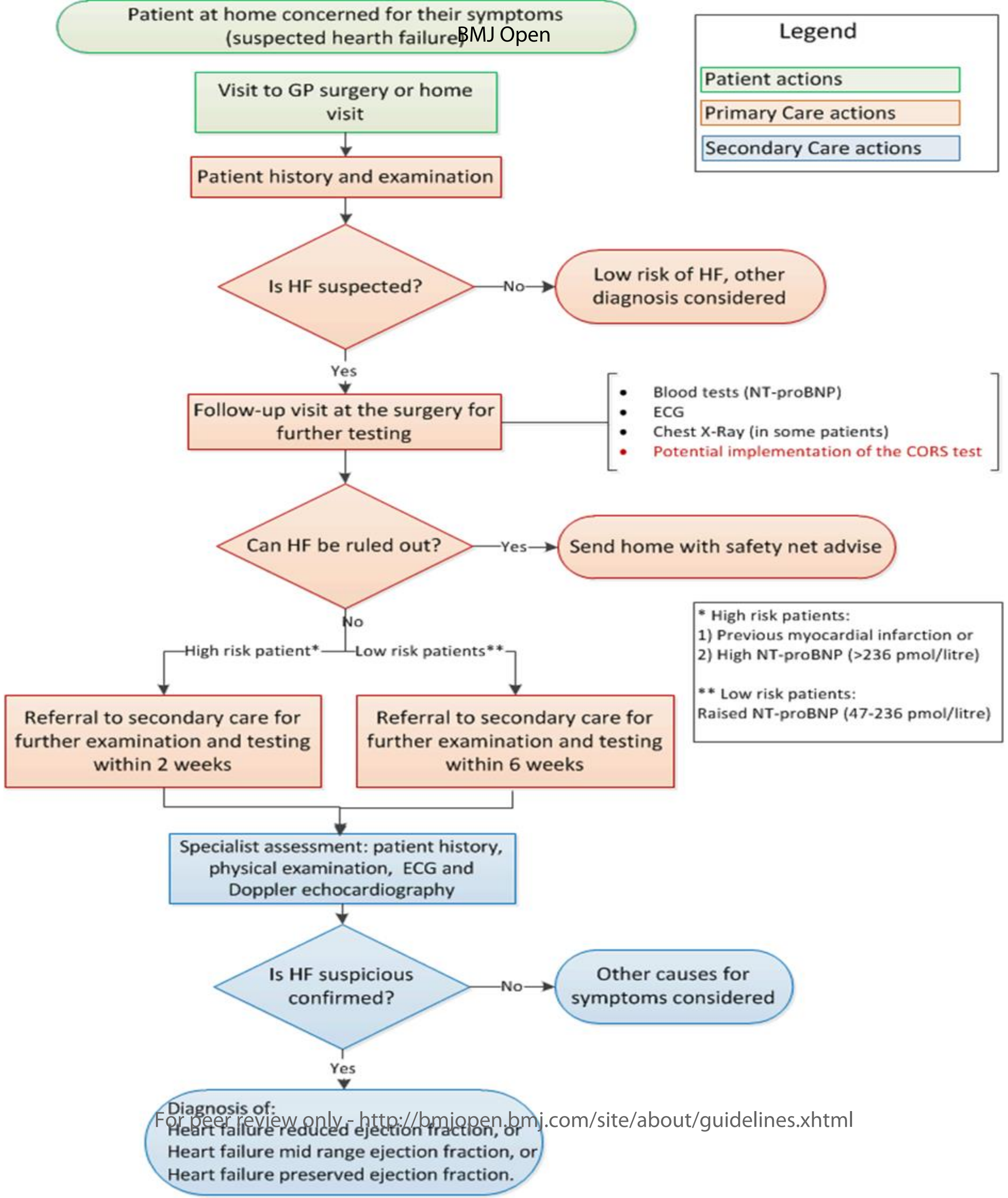
Patient at home concerned for their symptoms (suspected hearth failure)
Legend
Patient actions
Primary Care actions
Secondary Care actions
Visit to GP surgery or home visit
Patient history and examination
Is HF suspected?
No
Low risk of HF, other diagnosis considered
Yes
Follow-up visit at the surgery for further testing
• Blood tests (NT-proBNP)
• ECG
• Chest X-Ray (in some patients)
• Potential implementation of the CORS test
Can HF be ruled out?
Yes
Send home with safety net advise
No
High risk patient*
Low risk patients**
* High risk patients:
1) Previous myocardial infarction or
2) High NT-proBNP (>236 pmol/litre)
** Low risk patients:
Raised NT-proBNP (47-236 pmol/litre)
Referral to secondary care for further examination and testing within 2 weeks
Referral to secondary care for further examination and testing within 6 weeks
Specialist assessment: patient history, physical examination, ECG and Doppler echocardiography
Is HF suspicious confirmed?
No
Other causes for symptoms considered
Yes
Diagnosis of:
Heart failure reduced ejection fraction, or
Heart failure mid range ejection fraction, or
Heart failure preserved ejection fraction.

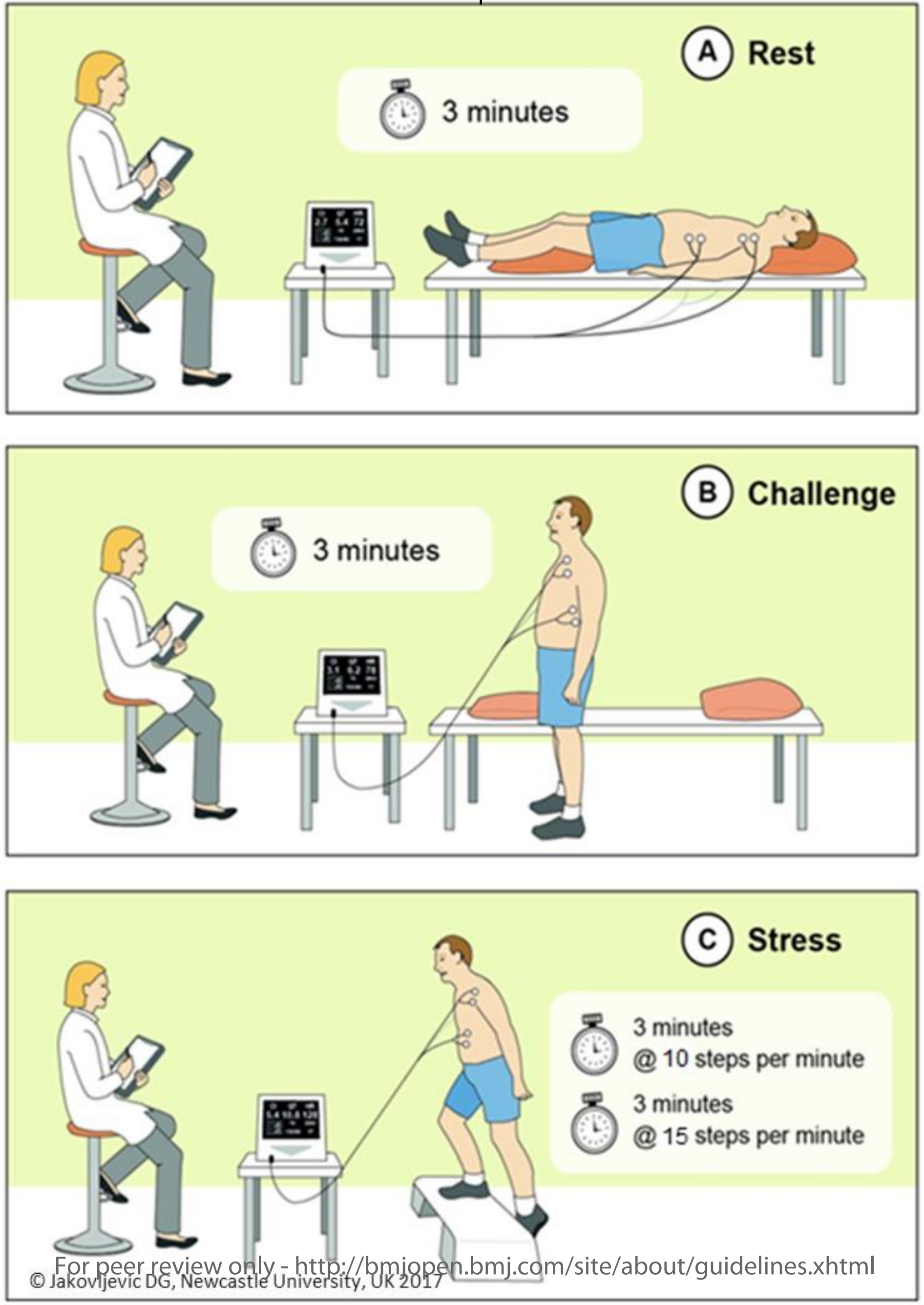


For peer review only - http://bmjopen.bmj.com/site/about/guidelines.xhtml

**Interview Schedule v1:** Consultant Cardiologists
**Project Title:** Confirmation of Acceptability of a Novel Cardiac Output Response to Stress (CORS) Test to Improve Diagnosis of Heart Failure in Primary Care

**PART 1**

**CLINICAL CARE PATHWAY**

What is the clinical care pathway for a patient with a suspected heart failure diagnosis in primary care?

When is a patient referred to secondary care?

Do the referral times from suspected heart failure diagnosis in primary care to secondary care need to be improved?

Prompt: if so, what needs to be improved? Strategy for improvement

What is your role in the clinical care pathway for a patient with a suspected heart failure diagnosis?

During a heart failure diagnostic clinic, how many suspected cases of heart failure do you typically see?

How many of these cases lead to diagnosis of heart failure?

Does the current clinical care pathway lead to you seeing many patients who are unlikely to receive a heart failure diagnosis in secondary care?

Prompt: If so how can this be issue be addressed?

How long does it take to determine a suspected heart failure case in secondary care?

Do the referral times from suspected heart failure diagnosis in primary care to confirmed diagnosis need to be improved?

Prompt: if so what needs to change?

For peer review only

## VIDEO OF CARDIAC OUTPUT RESPONSE TO STRESS (CORS) TEST

We are proposing a novel test which has been demonstrated to improve suspected heart failure diagnosis. This should therefore improve the number of patient referrals to secondary care.

## PART 2

## NEW CORS TEST

This novel test has been demonstrated to improve suspected heart failure diagnosis. This should therefore improve the number of patient referrals to secondary care.

What are your thoughts about this potential improvement in diagnosis being implemented in the clinical care pathway for heart failure?

Prompt: design, suitability and access to patients

What is your understanding of the new test?

Was there any information in the demonstration video that was difficult to understand?

Would a patient with a suspected heart failure diagnosis be able to complete this test?

Prompt: possible challenges to patients, Physical ability, other health problems

Where in the clinical care pathway for suspected heart failure diagnosis would you expect this test to be performed?

Would you expect this test to be performed in primary or secondary care?

Prompt: advantages/disadvantages; access to test, costs, practical implications

If this test was to become a routine part of clinical care for a patient with a suspected heart failure diagnosis, who would you expect to deliver it in primary care?

Prompt: rational for opinion

If this test was to become a routine part of clinical care for a patient with a suspected heart failure diagnosis, who would you expect to deliver it in secondary care?

Prompt: rational for opinion

Who has the greater capacity to run this test as a routine part of clinical care for a patient with a suspected heart failure diagnosis?

What problems or difficulties do you think a healthcare professional (e.g. practice nurse) running this test may encounter with a patient performing this test?

What else might be needed to deliver this test in either primary or secondary care?

What training would be needed to be able to run this test efficiently?

Do you have any further comments about running this test as part of your routine clinical care for a patient with a suspected heart failure diagnosis?

For peer review only

**Interview Schedule v1:** General Practitioners (Primary Care)

**Project Title:** Confirmation of Acceptability of a Novel Cardiac Output Response to Stress (CORS) Test to Improve Diagnosis of Heart Failure in Primary Care

**PART 1**

**CLINICAL CARE PATHWAY**

What is the clinical care pathway for a patient with a suspected heart failure diagnosis in primary care?

What is your role in the clinical care pathway for a patient with a suspected heart failure diagnosis?

What tests are performed to determine risk of heart failure?

Who would perform these tests in your practice?

How many times is a patient seen before they are referred to secondary care with suspected heart failure diagnosis?

How long is a typical consultation with a patient with a suspected heart failure diagnosis?

Who would inform a patient who was on the clinical care pathway for suspected heart failure diagnosis?

**REFERRAL**

When is a patient referred to secondary care?

What is your role in the referral process?

What are your thoughts on the referral time to secondary care?

When do you expect to receive the outcome from the referral?

What are the next steps after a patient has been diagnosed with heart failure?

What is your role in the continuing care of a patient diagnosed with heart failure?

Do the referral times from suspected heart failure diagnosis in primary care to confirmed diagnosis need to be improved?

Prompt: what needs improving? Strategy for improvement?

**VIDEO OF CARDIAC OUTPUT RESPONSE TO STRESS (CORS) TEST**

We are proposing a novel test which has been demonstrated to improve suspected heart failure diagnosis. This should therefore improve the number of patient referrals to secondary care

For peer review only

**PART 2**

**NEW CORS TEST**

This novel test has been demonstrated to improve suspected heart failure diagnosis. This should therefore improve the number of patient referrals to secondary care.

What are your thoughts about this potential improvement in diagnosis being implemented in the clinical care pathway for heart failure?

What is your understanding of the new test?

Was there any information in the demonstration video that was difficult to understand?

Would a patient with a suspected heart failure diagnosis be able to complete this test?

Could this test be added into a routine consultation for suspected heart failure diagnosis? Or would you expect the patient need to attend a separate appointment to complete the test?

Do you anticipate any immediate barriers to implementing this test as part of your routine clinical care for a patient with a suspected heart failure diagnosis in your practice?

Prompt: How easy would it be to implement this test in your practice? Are there challenges?

If this test was to become a routine part of clinical care for a patient with a suspected heart failure diagnosis, who would you expect to deliver it?

Prompt: rational for opinion

What problems or difficulties do you think you (or the nominated colleague, i.e. practice nurse) may encounter when using this test?

What else might you need to deliver this test in practice?

Prompt: Training needs

How likely is it that this test will become part of routine clinical care for a patient with a suspected heart failure diagnosis?

Prompt: Diagnostic urgency, suitability for patients, convenience

Where in the clinical care pathway for suspected heart failure diagnosis would you anticipate this test being implemented?

Prompt: Rational for opinion

Where do you think this test would be delivered in your practice?

Prompt: rational for opinion

Do you have the capacity to run this test as a routine part of clinical care for suspected heart failure diagnosis?

Do you have any further comments about running this test as part of your routine clinical care for a patient with a suspected heart failure diagnosis?

For peer review only

**Interview Schedule v1:** Practice Nurse (Primary Care)

**Project Title:** Confirmation of Acceptability of a Novel Cardiac Output Response to Stress (CORS) Test to Improve Diagnosis of Heart Failure in Primary Care

**PART 1**

**CLINICAL CARE PATHWAY**

What is the clinical care pathway for a patient with a suspected heart failure diagnosis in primary care?

What is your role in the clinical care pathway for a patient with a suspected heart failure diagnosis?

What tests are performed to determine risk of heart failure?

Who would perform these tests in your practice?

How long do the tests typically take?

How many times is a patient seen before they are referred to secondary care with a suspected heart failure diagnosis?

How long is a typical suspected heart failure diagnosis consultation with yourself?

Who would inform a patient who was on the clinical care pathway for suspected heart failure?

**REFERRAL**

When is a patient referred to secondary care?

Are you involved in the referral process?

What are your thoughts on the referral time to secondary care?

What are the next steps after a patient has been diagnosed with heart failure?

What is your role in the continuing care of a patient diagnosed with heart failure?

For peer review only

**VIDEO OF CARDIAC OUTPUT RESPONSE TO STRESS (CORS) TEST**

We are proposing a novel test which has been demonstrated to improve suspected heart failure diagnosis. This should therefore improve the number of patient referrals to secondary care

## PART 2

## NEW CORS TEST

This novel test has been demonstrated to improve suspected heart failure diagnosis. This should therefore improve the number of patient referrals to secondary care.

What are your thoughts about this potential improvement in diagnosis being implemented in the clinical care pathway for heart failure?

Prompt: What is your understanding of the new test?

Was there any information in the demonstration video that was difficult to understand?

Would a patient with a suspected heart failure diagnosis be able to complete this test?

Prompt experience of working with heart failure patients

Could this test be added into a routine consultation for suspected heart failure diagnosis? Or would you expect the patient need to attend a separate appointment to complete the test?

Prompt logistics and timing, space, staff availability.

Do you anticipate any immediate barriers to implementing this test as part of your routine clinical care for a patient with a suspected heart failure diagnosis?

How easy do you think it will be to implement this test in practice?

If this test was to become a routine part of clinical care for a patient suspected with a heart failure diagnosis, who would you expect to deliver it?

Prompt: rational for suggestion

What problems or difficulties do you think you may encounter when using this test?

What problems or difficulties do you think you may encounter with patients performing this test?

What else might you need to deliver this test in practice?

What training would you (or a colleague) need to be able to run this test?

How confident would you be about running this test?

How likely is it that this test will become part of routine clinical care for a patient with a suspected heart failure diagnosis?

Where in the clinical care pathway for suspected heart failure diagnosis would you anticipate this test being implemented?

Prompt: rational for opinion

For peer review only

Where do you think this test would be delivered in your practice?

Do you have any further comments about running this test as part of your routine clinical care for a patient with a suspected heart failure diagnosis?

For peer review only

**Interview Schedule v1:** Practice Manager (Primary Care)

**Project Title:** Confirmation of Acceptability of a Novel Cardiac Output Response to Stress (CORS) Test to Improve Diagnosis of Heart Failure in Primary Care

**PART 1**

**CLINICAL CARE PATHWAY**

Who would perform these tests in your practice?

How many times is a patient seen before they are referred to secondary care with suspected heart failure diagnosis?

How long is a typical consultation with a patient with a suspected heart failure diagnosis?

**REFERRAL**

A patient with a moderate to high NT-proBNP reading requires assessment by a specialist and echocardiogram in a heart failure diagnostic clinic in secondary care.

What is your role in the referral process?

What are your thoughts on the referral time to secondary care?

When do you expect to receive the outcome from the referral?

What are the next steps after a patient has been diagnosed with heart failure?

Do the referral times from suspected heart failure diagnosis in primary care to confirmed diagnosis need to be improved?

For peer review only

**VIDEO OF CARDIAC OUTPUT RESPONSE TO STRESS (CORS) TEST**

We are proposing a novel test which has been demonstrated to improve suspected heart failure diagnosis. This should therefore improve the number of patient referrals to secondary care

## PART 2

## NEW CORS TEST

This novel test has been demonstrated to improve suspected heart failure diagnosis. This should therefore improve the number of patient referrals to secondary care.

What are your thoughts about this potential improvement in diagnosis being implemented in the clinical care pathway for heart failure?

Prompt: is this test necessary?

What is your understanding of the new test?

Was there any information in the demonstration video that was difficult to understand?

Would you expect commissioners to supply this equipment for each practice?

Prompt: who carries the burden for implementation?

What are your thoughts on the cost of this test per patient?

How easy would it be to implement this test in your practice?

Do you have the staff capacity to run this test as a routine part of clinical care for suspected heart failure diagnosis?

If this test was to become a routine part of clinical care for a patient with a suspected heart failure diagnosis, who would you expect to deliver it?

Prompt: rational for opinion

What problems or difficulties do you think the nominated colleague, e.g practice nurse may encounter when using this test?

What else might you need to deliver this test in practice?

What training would the nominated colleague, i.e. practice nurse need to be able to run this test?

Where do you think this test would be delivered in your practice?

Do you have any further comments about this test becoming integrated into routine clinical care for a patient with a suspected heart failure diagnosis?

For peer review only

# COREQ (COnsolidated criteria for REporting Qualitative research) Checklist

A checklist of items that should be included in reports of qualitative research. You must report the page number in your manuscript where you consider each of the items listed in this checklist. If you have not included this information, either revise your manuscript accordingly before submitting or note N/A.

| Topic | Item No. | Guide Questions/Description | Reported on Page No. |
|---|---|---|---|
| **Domain 1: Research team and reflexivity** | | | |
| *Personal characteristics* | | | |
| Interviewer/facilitator | 1 | Which author/s conducted the interview or focus group? | 5 |
| Credentials | 2 | What were the researcher's credentials? E.g. PhD, MD | 5 |
| Occupation | 3 | What was their occupation at the time of the study? | 5 |
| Gender | 4 | Was the researcher male or female? | 5 |
| Experience and training | 5 | What experience or training did the researcher have? | 5 |
| *Relationship with participants* | | | |
| Relationship established | 6 | Was a relationship established prior to study commencement? | 5 |
| Participant knowledge of the interviewer | 7 | What did the participants know about the researcher? e.g. personal goals, reasons for doing the research | n/a |
| Interviewer characteristics | 8 | What characteristics were reported about the inter viewer/facilitator? e.g. Bias, assumptions, reasons and interests in the research topic | n/a |
| **Domain 2: Study design** | | | |
| *Theoretical framework* | | | |
| Methodological orientation and Theory | 9 | What methodological orientation was stated to underpin the study? e.g. grounded theory, discourse analysis, ethnography, phenomenology, content analysis | 5,6 |
| *Participant selection* | | | |
| Sampling | 10 | How were participants selected? e.g. purposive, convenience, consecutive, snowball | 5 |
| Method of approach | 11 | How were participants approached? e.g. face-to-face, telephone, mail, email | 5 |
| Sample size | 12 | How many participants were in the study? | 14 |
| Non-participation | 13 | How many people refused to participate or dropped out? Reasons? | n/a |
| *Setting* | | | |
| Setting of data collection | 14 | Where was the data collected? e.g. home, clinic, workplace | 5 |
| Presence of non-participants | 15 | Was anyone else present besides the participants and researchers? | no |
| Description of sample | 16 | What are the important characteristics of the sample? e.g. demographic data, date | n/a |
| *Data collection* | | | |
| Interview guide | 17 | Were questions, prompts, guides provided by the authors? Was it pilot tested? | 6 |
| Repeat interviews | 18 | Were repeat inter views carried out? If yes, how many? | no |
| Audio/visual recording | 19 | Did the research use audio or visual recording to collect the data? | 5 |
| Field notes | 20 | Were field notes made during and/or after the inter view or focus group? | No |
| Duration | 21 | What was the duration of the inter views or focus group? | 6 |
| Data saturation | 22 | Was data saturation discussed? | n/a |
| Transcripts returned | 23 | Were transcripts returned to participants for comment and/or | no |

| Topic | Item No. | Guide Questions/Description | Reported on Page No. |
|---|---|---|---|
| | | correction? | |
| **Domain 3: analysis and findings** | | | |
| *Data analysis* | | | |
| Number of data coders | 24 | How many data coders coded the data? | 6 |
| Description of the coding tree | 25 | Did authors provide a description of the coding tree? | No |
| Derivation of themes | 26 | Were themes identified in advance or derived from the data? | No |
| Software | 27 | What software, if applicable, was used to manage the data? | n/a |
| Participant checking | 28 | Did participants provide feedback on the findings? | No |
| *Reporting* | | | |
| Quotations presented | 29 | Were participant quotations presented to illustrate the themes/findings? Was each quotation identified? e.g. participant number | 7-13 |
| Data and findings consistent | 30 | Was there consistency between the data presented and the findings? | 7-13 |
| Clarity of major themes | 31 | Were major themes clearly presented in the findings? | 7-13 |
| Clarity of minor themes | 32 | Is there a description of diverse cases or discussion of minor themes? | No |

Developed from: Tong A, Sainsbury P, Craig J. Consolidated criteria for reporting qualitative research (COREQ): a 32-item checklist for interviews and focus groups. *International Journal for Quality in Health Care*. 2007. Volume 19, Number 6: pp. 349 – 357

**Once you have completed this checklist, please save a copy and upload it as part of your submission. DO NOT include this checklist as part of the main manuscript document. It must be uploaded as a separate file.**

For peer review only
